# Piceatannol Attenuates Benzo[*a*]pyrene/DSS-Induced Colorectal Cancer in Mice via Modulation of Gut Microbiota and Inhibition of the PI3K/AKT/mTOR Pathway

**DOI:** 10.1021/acs.jafc.5c05807

**Published:** 2025-09-25

**Authors:** Pin-Yu Ho, Yen-Chun Koh, Wei-Sheng Lin, Chin-Jui Ho, Anju Majeed, Chi-Tang Ho, Min-Hsiung Pan

**Affiliations:** a Institute of Food Science and Technology, 33561 National Taiwan University, Taipei 10617, Taiwan; b Department of Food Science, 252857 National Quemoy University, Quemoy County 89250, Taiwan; c School of Pharmacy, College of Medicine, 33561 National Taiwan University, Taipei 100025, Taiwan; d Program in Neuroscience, Baylor College of Medicine, Houston, Texas 77030, United States; e Sami-Sabinsa Group Limited, Bengaluru, Karnataka 560058, India; f Department of Food Science, Rutgers University, New Brunswick, New Jersey 08901-8520, United States; g Department of Public Health, China Medical University, Taichung 40402, Taiwan; h Department of Health and Nutrition Biotechnology, Asia University, Taichung City 413305, Taiwan

**Keywords:** colorectal cancer, piceatannol, gut microbiota, inflammation

## Abstract

Benzo­[*a*]­pyrene (B­[a]­P) promotes colorectal cancer (CRC) under chronic inflammation. Piceatannol (PIC), a polyphenol with anti-inflammatory properties, was investigated for its timing-dependent effects in a B­[a]­P/dextran sulfate sodium (DSS)-induced CRC mouse model. ICR mice received B­[a]P and DSS, followed by PIC intervention at different time points. Continuous PIC treatment (PI group) significantly reduced tumor burden, disease activity index, and intestinal permeability. Proinflammatory cytokines significantly decreased, whereas interleukin-10 increased. RNA-seq analysis showed that PIC upregulated transglutaminase 3 (Tgm3), which was associated with suppression of the phosphoinositide 3-kinase/AKT/mechanistic target of rapamycin pathway, downregulation of proinflammatory and tumor-promoting genes, and enhancement of epithelial repair genes. Gut microbiota analysis demonstrated restored microbial diversity, characterized by increased *Roseburia faecis* and *Kineothrix alysoides* and a reduction in *Turicibacter sanguinis* and *Romboutsia ilealis*. Continuous piceatannol administration attenuates CRC through microbiota and inflammatory regulation, with Tgm3 upregulation potentially contributing to these effects.

## Graphical abstract

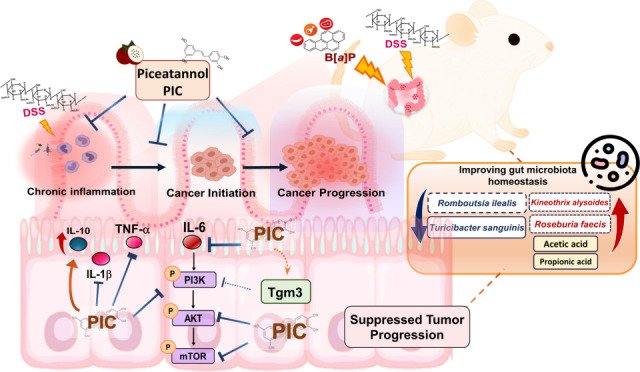

## Highlights

1. Early PIC intervention maximizes tumor suppression and gut microbiota restoration.
2. PIC attenuates CRC progression by inhibiting the PI3K/AKT/mTOR pathway, accompanied by Tgm3 upregulation.
3. PIC reshapes gut microbiota by reducing proinflammatory taxa and enhancing SCFA production.

## 1 Introduction

Colorectal cancer (CRC) is a leading cause of cancer-related mortality worldwide, with increasing incidence linked to environmental carcinogens and chronic inflammation.^1^ Among these carcinogens, benzo­[*a*]­pyrene (B­[a]­P), a polycyclic aromatic hydrocarbon commonly found in grilled foods, cigarette smoke, and air pollution, has been identified as a potent mutagen that promotes tumorigenesis.^2–4^ Inflammatory conditions further exacerbate CRC risk, with dextran sulfate sodium (DSS) frequently used to model colitis-associated CRC in experimental studies.^5,6^

Piceatannol (PIC), a naturally occurring polyphenol found in grapes, passion fruit, and other plant sources, has demonstrated anti-inflammatory and anticancer properties.^7^ Previous studies suggest that PIC exerts protective effects against various cancers by modulating immune responses, reducing oxidative stress, and regulating signaling pathways such as phosphoinositide 3-kinase (PI3K)/protein kinase B (AKT)/mechanistic target of rapamycin (mTOR).^8^ For example, PIC was reported to alleviate cisplatin-induced ovarian toxicity through PI3K/AKT pathway modulation,^9^ and a combined extract of PIC and *Adiantum capillus-veneris* demonstrated antiproliferative activity in a hydrazine-induced CRC model.^10^ Mechanistically, PIC is a hydroxylated analog of resveratrol (RES) with an additional 3′-hydroxyl group (3,3′,4′,5′-tetrahydroxystilbene) and distinct metabolism and bioactivity than RES, which may contribute to distinct bioactivities.^11^ Especially, colon-targeted delivery of PIC enhances anticolitic efficacy while inhibiting nuclear factor kappa B (NF-κB) and activating hypoxia-inducible factor-1 (HIF-1)/nuclear factor erythroid 2-related factor 2 (Nrf2) in the colon.^12^ In DSS colitis, PIC also preserves tight junction integrity and reshapes the gut microbiota with enrichment of butyrate-producing taxa.^13^ However, these studies did not address PIC’s effects in inflammation-associated CRC models nor explore its impact on gut microbiota and host transcriptomic responses. The specific role of PIC in modulating the gut microenvironment and molecular signaling in colitis-associated CRC remains largely unexplored.

The tumor microenvironment (TME) plays a critical role in CRC progression, with inflammatory cytokines, matrix metalloproteinases (MMPs), and epithelial-mesenchymal transition (EMT)-associated factors contributing to tumor invasion.^14^ The PI3K/AKT/mTOR signaling pathway, frequently dysregulated in CRC, is a key regulator of cell proliferation and survival.^15^ Additionally, gut microbiota composition significantly influences inflammation and CRC development, with certain bacterial taxa contributing to tumor-promoting conditions through metabolic and immune interactions.^16^

This study investigates the chemopreventive effects of PIC in a B­[a]­P/DSS-induced CRC mouse model, focusing on timing-dependent intervention. We hypothesize that early and sustained PIC administration will more effectively reduce tumor burden, modulate inflammatory responses, and restore gut microbiota balance compared to delayed interventions. To elucidate these mechanisms, we conducted RNA sequencing (RNA-seq), gut microbiota analysis, and Western blot validation to assess the impact of PIC on gene expression, microbial diversity, and PI3K/AKT/mTOR pathway activity. These findings provide novel insights into the therapeutic potential of PIC in inflammation-associated CRC prevention and highlight the significance of intervention timing.

## 2 Materials and Methods

### 2.1 Materials

PIC (>95%, determined by high-performance liquid chromatography) was supplied by Sabinsa Corporation (East Windsor, NJ, USA). Benzo­[*a*]­pyrene (B­[a]­P), β-actin, and fluorescein isothiocyanate (FITC)-dextran were obtained from Sigma-Aldrich Chemical Co. (St. Louis, MO, USA). Dextran sulfate sodium (DSS) was purchased from MP Biomedicals, LLC, Illkirch, France. Primary antibodies against phosphoinositide 3-kinase (PI3K) and phosphorylated PI3K (p-PI3K) were obtained from Santa Cruz Biotechnology (Dallas, TX, USA), while antibodies against protein kinase B (Akt) and phosphorylated Akt (p-Akt; Tyr308) were acquired from Cell Signal Technology (Beverly, MA, USA). Transglutaminase 3 (Tgm3) antibodies were from Thermo Fisher Scientific (Waltham, MA, USA). Secondary antibodies were acquired from GeneTex (Irvine, CA, USA) and Abcam PLC (Cambridge, UK). The Bio-Rad protein assay dye reagent used for protein quantification was obtained from Bio-Rad Laboratories (Munich, Germany).

### 2.2 Animals and Experimental Design

Sixty male ICR (Institute of Cancer Research) mice, aged 4 weeks and weighing approximately 20 ± 2 g, were procured from BioLASCO Taiwan Co. (Taipei, Taiwan). Mice were housed in an environmentally controlled facility maintained at 23 °C with 50% relative humidity and a 12 h light/dark cycle. Food and water were provided ad libitum. All experimental procedures involving animals were performed in accordance with the Laboratory Animal Care Guidelines of the Taiwan Ministry of Agriculture, and the protocol was approved by the Institutional Animal Care and Use Committee (IACUC) of National Taiwan University (Approval Number NTU-109-EL-00180), ensuring ethics compliance without encountering unexpected or unusually high security issues.

A CRC induction model, adapted from Hakura et al.,^17^ was used. Mice aged 4–5 weeks received B­[a]P at a dose of 125 mg/kg/day via daily gavage for five consecutive days. After a one-week withdrawal period, CRC was induced using 2% DSS in drinking water, administered in two cycles at weeks 3 and 6. Following a one-week acclimatization period, mice were randomly assigned to five groups (*n* = 12 per group) for an 18-week experimental duration: the control group (Cont) received a standard diet with deionized water, the induction group (B­[a]­P/DSS) received a standard diet with 2% DSS in drinking water, the PI group (B­[a]­P/DSS + 0.025% PIC, weeks 1–18) received continuous PIC treatment, the PII group (B­[a]­P/DSS + 0.025% PIC, weeks 1–9) received early PIC intervention, and the PIII group (B­[a]­P/DSS + 0.025% PIC, weeks 9–18) received late PIC intervention.

The dosage of PIC used in this study (0.025%) was based on previous work from our laboratory.^13^ PIC was uniformly mixed into the feed to ensure consistent intake. DSS was administered orally in two cycles, with two-week intervals of normal drinking water between treatments. The control group received only deionized water throughout the study.

### 2.3 Disease Activity Index

The disease activity index (DAI) is a well-established indicator of colorectal inflammation in DSS-induced inflammatory bowel disease (IBD) models and correlates with pathological characteristics, as shown in Table S2.^18^ During the DSS treatment period, DAI scores were recorded daily based on weight changes, stool consistency, and the presence of bloody stools. The DAI score was calculated using the following formula: DAI index = (bloody stool score + diarrhea score + weight loss score) ÷ 3.^19^

### 2.4 Intestinal Permeability Tests

Intestinal permeability was measured using FITC-dextran (4 kDa), a fluorescent tracer. A stock solution (125 mg/mL) was prepared by dissolving 500 mg of FITC-dextran in 4 mL of distilled water. One week before sacrifice, mice fasted for 4 h were gavaged with FITC-dextran (400 mg/kg body weight, ≤200 μL). Blood samples (200 μL) were collected 2 h postgavage from the tail vein and centrifuged at 10,000*g* for 10 min at 4 °C. Fluorescence intensity was quantified (excitation 485 nm, emission 528 nm) to determine intestinal permeability.

### 2.5 Serum Biochemical Analysis and Cytokine Quantification

Blood samples were collected from the mice and centrifuged at 1500*g* for 20 min at 4 °C. Separated serum was stored at −80 °C until analysis. Biochemical analyses were conducted at the National Laboratory Animal Center (NLAC) in Taipei, Taiwan, using a Hitachi 7080 biochemical analyzer (Hitachi, Tokyo, Japan), following the manufacturer’s instructions. Serum levels of alanine aminotransferase (ALT), aspartate aminotransferase (AST), creatinine (CREA), and blood urea nitrogen (BUN) were measured. Cytokine levels were quantified using ELISA kits from Elabscience Biotechnology Co., Ltd. (Wuhan, Hubei, China). Serum interleukin (IL)-10 levels were measured using kit E-UNEL-M0057, while intestinal tissue samples were analyzed for TNF-α (E-UNEL-M0103), IL-1β (E-UNEL-M0064), and IL-6 (E-UNEL-M0070).

### 2.6 Hematoxylin–Eosin Staining

Colon tissues were fixed in 10% formalin, embedded in paraffin, sectioned (3–5 μm), deparaffinized, rehydrated, and stained with hematoxylin and eosin (H&E) using reagents from Surgipath (Peterborough, UK). Microscopic examination was performed using an Olympus BX51 microscope (Olympus Corp., Tokyo, Japan), and pathological assessments were conducted by the NTUCM Laboratory Animal Center.

### 2.7 Histopathological Examination

Histological evaluations, including lesion identification, morphological analysis, and semiquantitative scoring using the colitis histological scoring system (Table S3),^20^ were performed by trained personnel at the NTUCM Experimental Animal Center under IACUC-approved protocols. Colon tissue and tumor samples were processed using standard H&E staining.

### 2.8 Western Blotting

Protein samples (25–35 μg) were mixed with a loading dye, denatured at 100 °C, and separated via SDS-PAGE. Proteins were transferred onto a polyvinylidene difluoride (PVDF) membrane (Millipore Corp., Bedford, MA, USA) at 450 mA for 4–6 h at 4 °C. Membranes were incubated with primary antibodies overnight (12 h, 4 °C), followed by secondary antibody incubation. Protein detection was performed using enhanced chemiluminescence and quantified using ImageJ software (National Institutes of Health, Bethesda, MD, USA).

### 2.9 Short-Chain Fatty Acid Analysis

The short-chain fatty acid (SCFA) content was analyzed using organic solvent extraction followed by gas chromatography–mass spectrometry (GC-MS) with an Agilent 7890 GC system and a DB-WAXetr column (Agilent Technologies, Inc., Santa Clara, CA, USA).^21^ Fecal samples (0.1 g) were homogenized in 0.5% phosphoric acid, centrifuged, extracted with ethyl acetate, and filtered before GC-MS injection.

### 2.10 Intestinal RNA Collection

Following euthanasia, intestinal tissues were photographed, measured, and washed with phosphate-buffered saline (PBS). Samples were preserved in RNAlater (Thermo Fisher Scientific, Waltham, MA, USA), and epithelial cells were scraped for RNA stabilization in a TRIzol reagent (Thermo Fisher Scientific, Waltham, MA, USA). Samples were stored at −80 °C for subsequent analysis.

### 2.11 RNA Sequencing and Analysis

RNA quality was assessed using a SimpliNano spectrophotometer (GE Healthcare Life Sciences, Chicago, IL, USA) and a Qsep 100 analyzer (BiOptic, Inc., New Taipei City, Taiwan). Transcriptome libraries were prepared using a KAPA mRNA HyperPrep Kit, followed by sequencing on an Illumina NovaSeq 6000 platform (Illumina, Inc., San Diego, CA, USA). Raw sequencing data were processed using Trimmomatic, HISAT2, FeatureCounts, DESeq2, and clusterProfiler for gene expression and pathway analysis.^22^

### 2.12 Microbial Analysis

Microbial community profiling was performed using 16S rRNA sequencing via next-generation sequencing (NGS) on the Illumina MiniSeq platform. Fecal DNA was extracted using an InnuPREP Stool DNA kit (Analytik Jena, Germany), amplified via PCR, and sequenced. Taxonomic classification was conducted using the Greengenes database to assess microbial diversity and composition.

### 2.13 Statistical Analysis

Statistical analyses were performed using one-way analysis of variance (ANOVA) followed by Duncan’s multiple range test. Data were analyzed using SPSS software (version 26.0.0; IBM Corp., Armonk, NY, USA). Results were considered statistically significant at *p* ≤ 0.05. All data are presented as means ± SEM.

## 3 Results

### 3.1 Effects of PIC Intervention Timing on the Appearance, Body Weight, DAI, Organ Weight, and Serum Biochemical Parameters in B­[a]­P/DSS-Induced CRC Mice

The experimental design is illustrated in Figure 1A. PIC administration at different stages significantly influenced the appearance, body weight, DAI, organ weight, and serum biochemical parameters in B­[a]­P/DSS-induced CRC mice. Appearance and body weight observations (Figure S1A and Figure 1B,C) showed that mice in the PI and PII groups maintained healthier body weights and exhibited less severe disease progression than those in the B­[a]­P/DSS group. Throughout the study, weight trends showed that the average body weight in the PI group remained consistently higher than in the B­[a]­P/DSS group, indicating that PIC administration mitigated weight loss associated with disease progression. Food and water intake values represent the average across the entire 18-week experimental period. Food intake and water consumption showed no significant differences among the groups (Table S1), suggesting that the observed effects were due to PIC intervention rather than differences in consumption.

**Figure 1:**
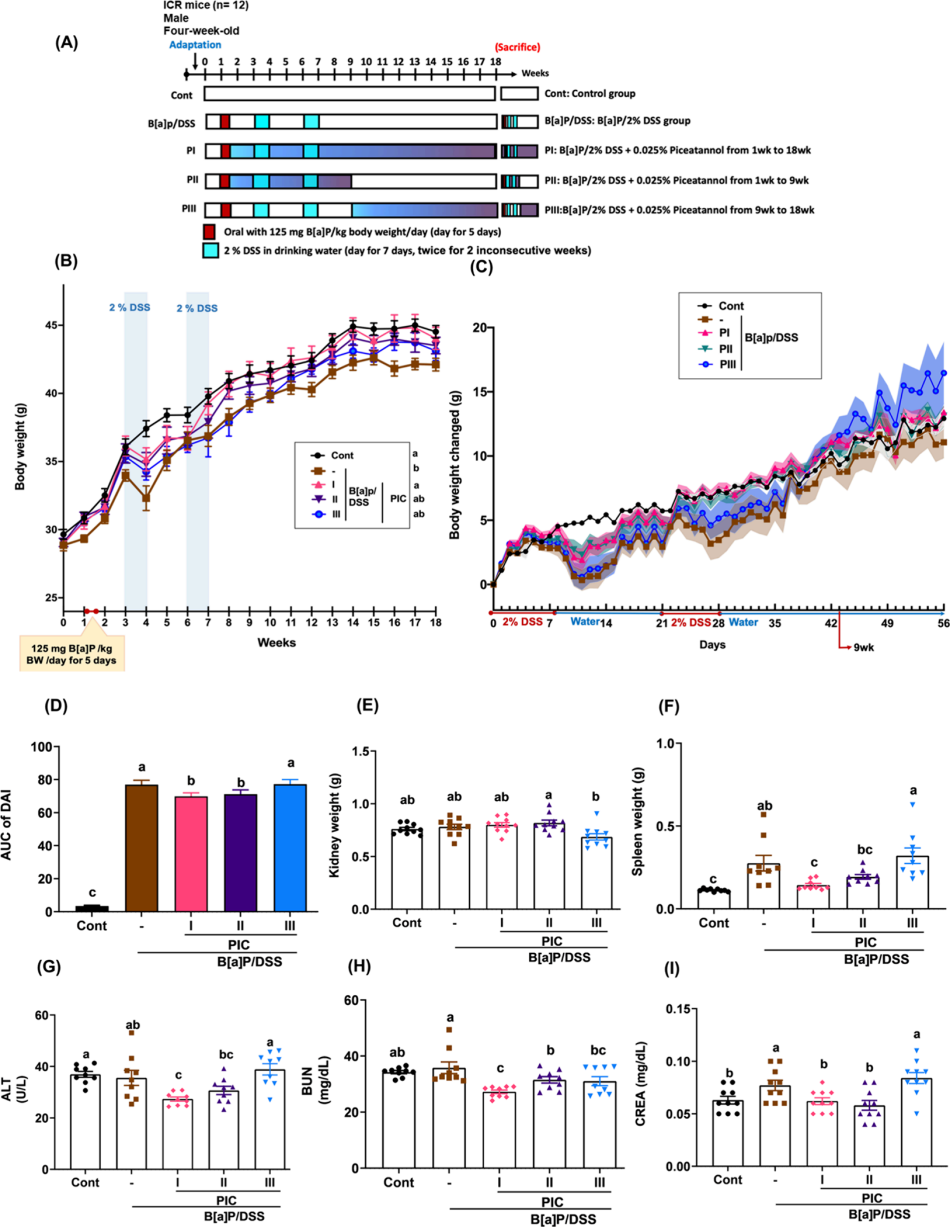
Effects of PIC on the body weight, DAI, organ, and serum biochemical parameters in B­[a]­P/DSS-induced colorectal cancer mice. (A) Experimental procedure. (B) Body weight changes within 18 weeks as recorded. (C) Body weight changed. (D) AUC of DAI score. (E) Kidney weight. (F) Spleen weight. (G) ALT. (H) BUN. (I) CREA. Data are presented as means ± SEM; *n* = 9–10 per group. *p*-values were determined by one-way ANOVA with subsequent Duncan’s multiple comparison test. The values with different letters (a, b, and c) are significantly different (*p* < 0.05) between each group. PI: B­[a]­P/2%DSS + 0.025% piceatannol from 1wk to 18wk; PII: B­[a]­P/2%DSS + 0.025% piceatannol from 1wk to 9wk; PIII: B­[a]­P/2%DSS + 0.025% piceatannol from 9wk to 18wk. 2% DSS was orally administered in deionized water for two cycles with a two-week rest between cycles. The control group (Cont) was given deionized water only.

The DAI, which assesses weight loss, diarrhea, and bloody stools, was significantly elevated during the induction phase and decreased following the switch to normal drinking water. The PI and PII groups displayed significantly lower DAI values compared to the B­[a]­P/DSS group (Figure S1B and Figure 1D), suggesting that PIC reduced inflammatory symptoms during CRC progression. Organ weight changes, which serve as sensitive indicators of toxicity, revealed no significant damage resulting from B­[a]­P/DSS exposure or PIC intervention. Figure S1C presents representative photographs of the liver, kidneys, and spleen for each group. Notably, liver weights remained consistent across all groups (Figure S1D), indicating no hepatotoxic effects. Kidney weights also showed no significant differences between the treatment and induction groups (Figure 1E). However, spleen weights were elevated in the B­[a]­P/DSS group, reflecting inflammation, which was effectively alleviated by PIC, especially in the PI group, showing that early stage intervention can help mitigate B­[a]­P/DSS-induced splenic macrophage infiltration (Figure 1F). Serum biochemical analyses further confirmed the nontoxic nature of the treatments. AST and ALT levels remained within normal ranges across all groups (Figure S2A and Figure 1G), with ALT levels in the PI group significantly lower than in other groups while still within the normal range, suggesting a potential protective effect of PIC on liver health. Kidney function, assessed by BUN and CREA levels, also remained within normal limits. Figure 1H,I shows that BUN and CREA levels in the PI group were lower than those in the B­[a]­P/DSS group. These findings highlight the chemopreventive potential of PIC, particularly when administered early and sustained throughout disease progression. PIC effectively mitigated B­[a]­P/DSS-induced colorectal carcinogenesis. Therefore, we sought to elucidate the molecular mechanisms underlying PIC’s protective effects, providing valuable insights for CRC prevention and treatment strategies.

### 3.2 PIC Improves Intestinal Morphology, Reduces Tumor Burden, and Modulates Inflammation in B­[a]­P/DSS-Induced CRC Mice

In the B­[a]­P/DSS-induced chronic enteritis and CRC model, significant alterations in intestinal morphology and inflammation were observed. Macroscopic examinations (Figure 2A) revealed shortened intestinal length, increased weight, and the presence of granulomatous lesions indicative of chronic inflammation and tumor development. These changes were accompanied by severe mucosal thickening and visible tumor lesions predominantly localized in the mid and distal colon of the B­[a]­P/DSS group. Quantitative analyses (Figure 2B–D) indicated that PIC intervention mitigated these pathological changes. The PI group exhibited significant improvements, including increased intestinal length, reduced intestinal weight, and enhanced barrier integrity, as evidenced by decreased FITC-dextran permeability. Additionally, tumor burden analyses (Figure 2E,F) showed that PIC markedly reduced the number and size of tumors, with the PI group demonstrating the most pronounced protective effects. This was further supported by histopathological findings (Table S4), revealing that the B­[a]­P/DSS group developed multiple adenomas (e.g., 2 preneoplastic lesions and 5 adenomas in mouse #1), indicating advanced tumor progression. In contrast, the PI group showed a notable reduction in tumor pathology, with some mice (e.g., mice #1 and #2) showing no detectable lesions, suggesting the protective potential of early PIC intervention. The PII and PIII groups displayed intermediate effects, with fewer adenomas compared to the B­[a]­P/DSS group but less pronounced benefits than the PI group. Histological evaluations (Figure 3A) confirmed that PIC-treated mice exhibited decreased mucosal thickening, reduced inflammatory cell infiltration, and improved tissue integrity compared to the B­[a]­P/DSS group. Colorectal tumor histological scores (Figure S2B–E and Figure 3B) further supported these findings, with significantly lower scores in the PI group, reflecting reduced tumor pathology. Supplementary histopathological data (Table S4) highlighted severe mucosal alterations in the B­[a]­P/DSS group, where adenomas and preneoplastic lesions were abundant. Inflammatory cytokine profiling (Figure 3C–F) revealed elevated levels of proinflammatory markers IL-1β, TNF-α, and IL-6 in the B­[a]­P/DSS group, consistent with epithelial barrier disruption and immune activation. Notably, PIC treatment reduced these proinflammatory cytokines while increasing anti-inflammatory IL-10 levels, suggesting its role in mitigating inflammation-driven tumorigenesis.

**Figure 2:**
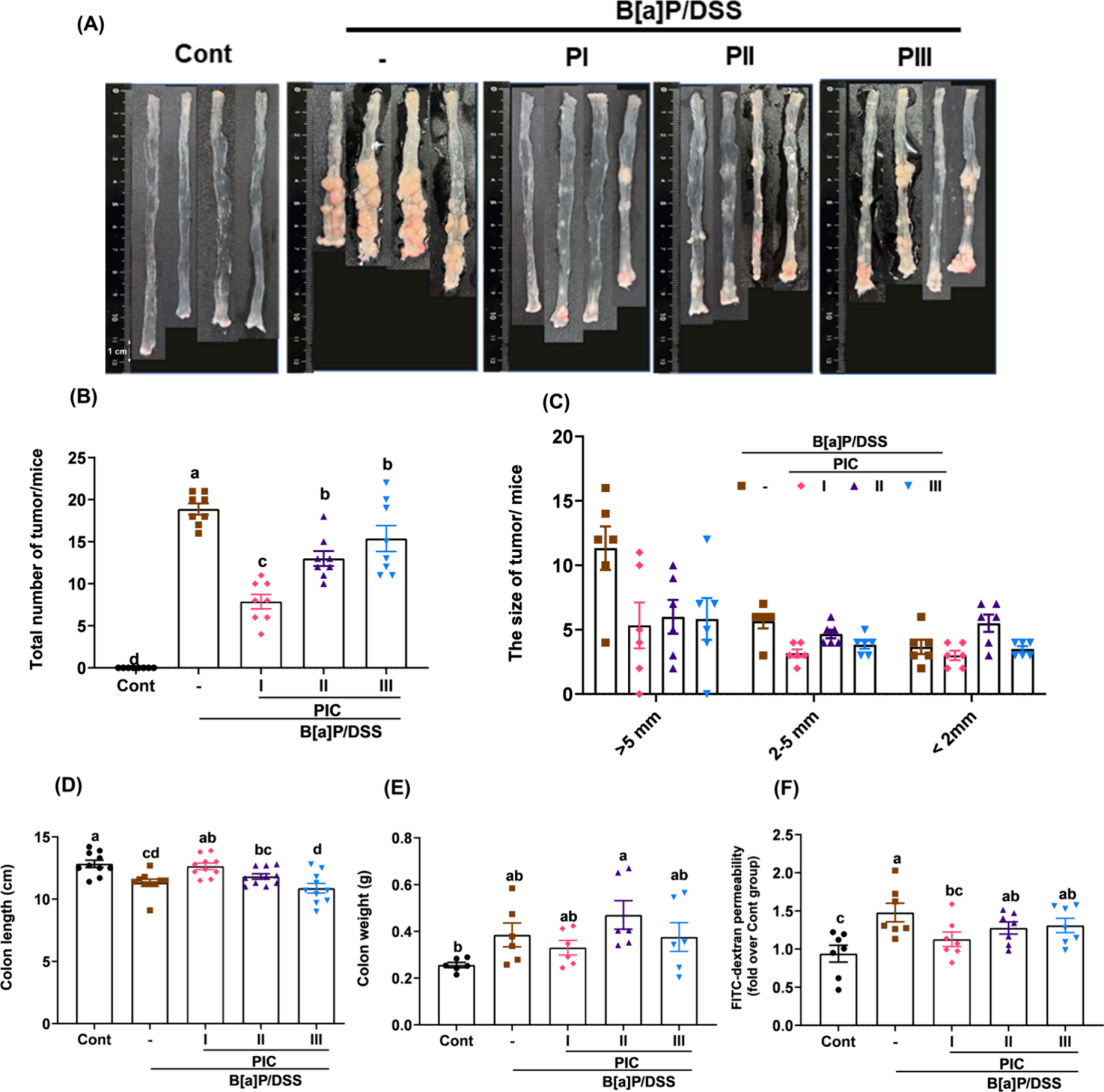
PIC alleviated B­[a]­P/DSS-induced colonic shortening and colon polyp number. (A) Macroscopic views of the mouse colons in each group. (B) Total number of tumors. (C) Size of tumors/mice. (D) Colon length. (E) Colon weight. (F) Gut permeability was assessed by measuring levels of FITC-dextran in serum after oral gavage of 4 kDa FITC-dextran for 4 h. Data are presented as means ± SEM; *n* = 3–8 per group. *p* values were determined by one-way ANOVA with subsequent Duncan’s multiple comparison test. The values with different letters (a, b, c, and d) are significantly different (*p* < 0.05) between each group.

**Figure 3:**
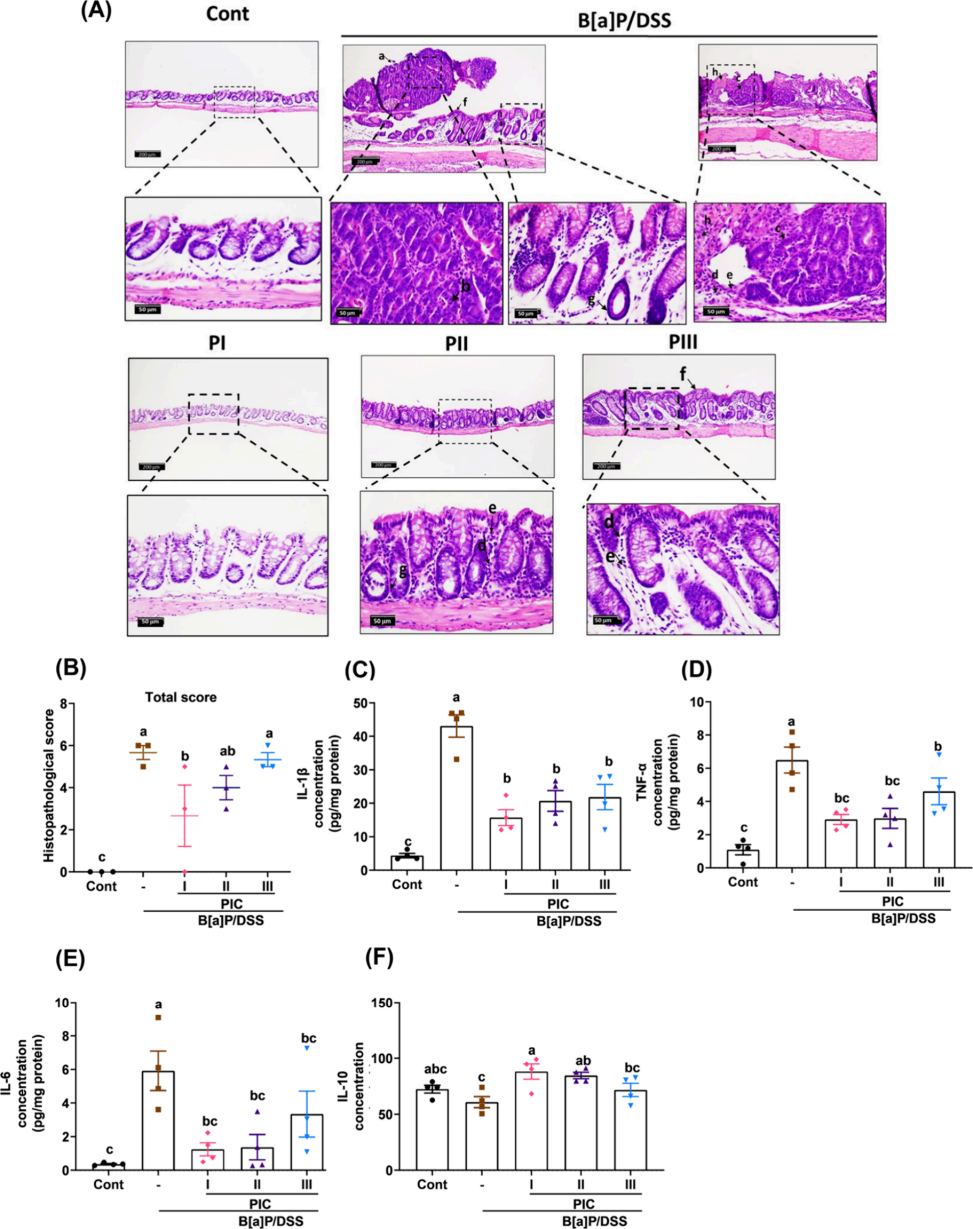
PIC attenuated B­[a]­P/DSS-induced colon tumorigenesis and inflammatory response. (A) Histopathology of mouse colonic mucosa from each group. The pedunculated tubular adenoma (arrow a) was composed of tubular structures lined by the dysplastic epithelium, which presented the absence of mucus secretion, cellular basophilia, loss of cellular polarity, hyperchromatic elongated nuclei with nuclear stratification, and numerous mitoses (arrow b). The preneoplastic lesions (arrow c) demonstrated focal epithelial dysplasia. The colon revealed slight infiltration of mononuclear cells (arrow d) and neutrophils (arrow e) in the mucosa and submucosa. Minimal mucosal hyperplasia (arrow f), slight loss of goblet cells (arrow g), and minimal granulation tissue formation (arrow h) were also detected (A, C: scale bars measure 200 μm; B, D, and E: scale bars measure 50 μm). Histopathological score of (B) total histopathological score. (C–E) TNF-α, IL-1β, and IL-6 in colonic tissues were determined by ELISA. (F) IL-10 in serum. Data are presented as means ± SEM; *n* = 3–4 per group. *p* values were determined by one-way ANOVA with subsequent Duncan’s multiple comparison test. The values with different letters (a, b, and c) are significantly different (*p* < 0.05) between each group.

### 3.3 RNA-Seq Analysis of Gene Expression Changes in the Intestinal Tissue of B­[a]­P/DSS-Induced CRC Mice Treated with PIC

RNA-Seq has surpassed microarray technology as the gold standard for gene expression studies.^23^ Principal component analysis (PCA) was used to evaluate the similarity within experimental groups. As shown in Figure 4A, the PI group clustered closely with the control group, indicating comparable sample characteristics. In contrast, the B­[a]­P/DSS treatment group exhibited significant divergence from the control. The volcano plot (Figure S3A) revealed significant upregulation of MMP genes, including *MMP10*, *MMP7*, *MMP13*, *MMP12*, *MMP8*, and *MMP3*, in the B­[a]­P/DSS treatment group compared to the control. Additionally, genes associated with the aryl hydrocarbon receptor (AhR) pathway (e.g., *Hsp90aa1*, *CYP1B1*, and *Arnt2*) and the Wnt signaling pathway (e.g., *Wnt10a* and *Wnt5a*) were upregulated. Proinflammatory cytokine genes *IL-6* and *IL-17a* were also markedly increased, reflecting an inflammatory microenvironment induced by B­[a]­P/DSS.

**Figure 4:**
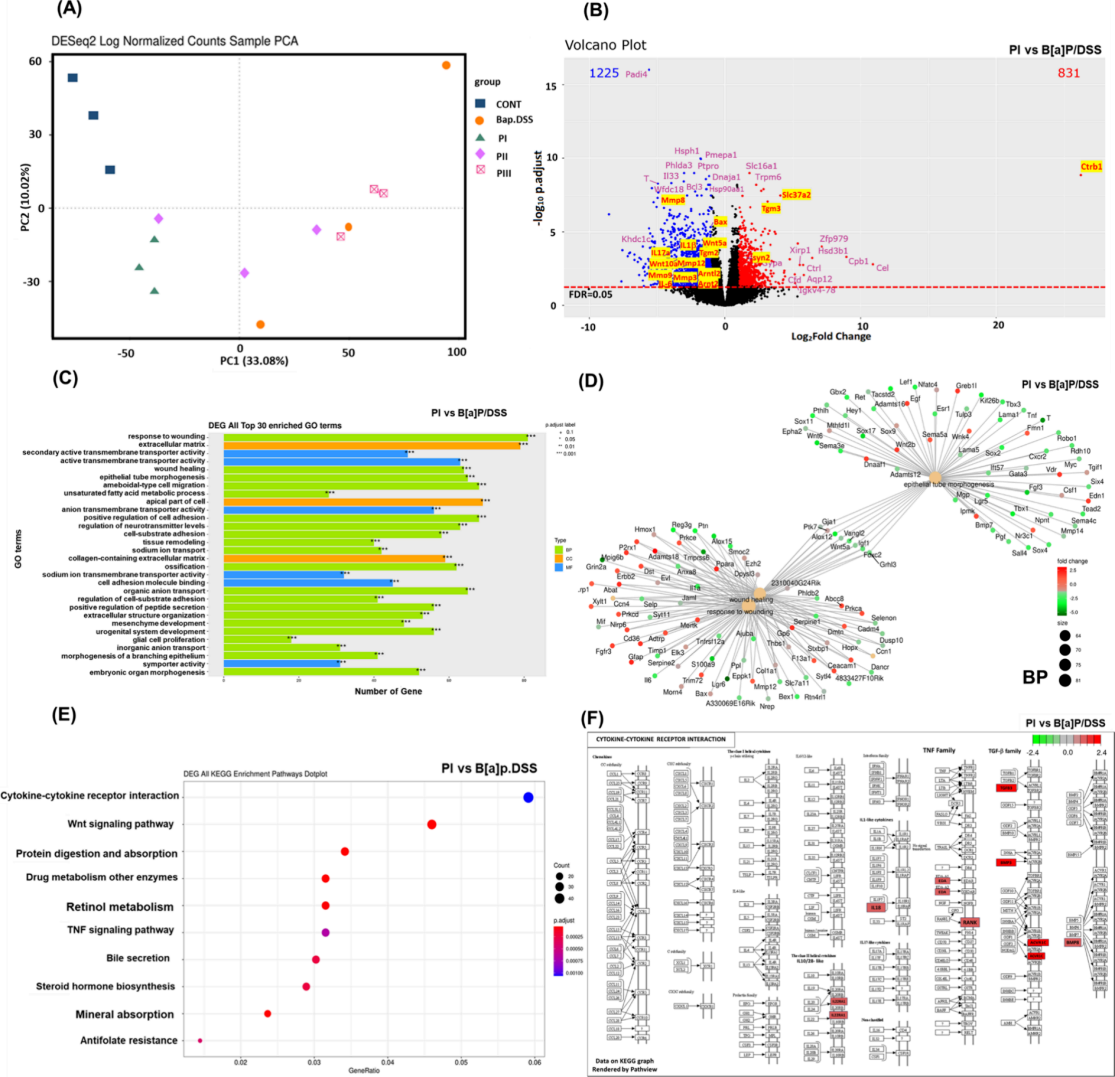
RNA-seq analysis and comparison of transcriptional responses and functional pathway alterations between the PI group and the induced group in B­[a]­P/DSS-induced colorectal cancer mice. (A) Principal component analysis. (B) Volcano plot of RNA sequencing data of genes between the PI group vs B­[a]­p/DSS group. Downregulated and upregulated genes are marked in blue and red, respectively, if they passed the threshold of the false discovery rate (FDR) < 0.05 and |log2FC| > 0.5. (C) Top 30 GO enrichment analysis in the PI vs B­[a]­P/DSS group. (D) GO gene-concept network of core genes between PI vs B­[a]­p/DSS in the biological process (BP). (E) KEGG pathway dot plot between the PI group and B­[a]­p/DSS group. (F) Cytokine–cytokine receptor interactions in the KEGG pathway view of the gene map between the PI group and B­[a]­p/DSS group. KEGG, Kyoto Encyclopedia of Genes and Genomes. The enriched genes will be marked on the reaction pathway diagram, with red representing positive regulation and green representing negative regulation. *n* = 3 per group.

Conversely, PIC treatment significantly downregulated MMP-related genes (*MMP9*, *MMP12*, *MMP8*, and *MMP3*), the AhR pathway transporter *Arnt2*, and proinflammatory cytokines (*IL-1*β and *IL-6*), as shown in Figure 4B. Moreover, PIC reduced the expression of Wnt signaling genes *Wnt10a* and *Wnt5a*, which are associated with tumor progression.^24^ Notably, *Ctrb1* (chymotrypsinogen B1) was upregulated in both the PI and PIII groups (Figure 4B and Figure S4B). As a key mediator of epithelial homeostasis, *Ctrb1* may contribute to the resolution of T cell-mediated colitis.^25^ Similarly, *Slc37a2* (solute carrier family 37 member 2) was upregulated in the PI and PII groups (Figure 4B and Figure S4A). *Slc37a2*, part of the solute carrier family 37, is involved in sugar–phosphate exchange and helps maintain glucose 6-phosphate activity, transport, and phosphate ion movement across membranes.^26^ It is located in the endoplasmic reticulum membrane and is essential to intestinal epithelial cells,^27^ linking nutrient and energy metabolism with immune activation. Research suggests that it acts as an early suppressor of inflammation, reducing macrophage activation and promoting resolution of acute inflammation.^28^

Another key finding was the upregulation of transglutaminase 3 (*Tgm3*) in the PI and PII groups. Tgm3 catalyzes the irreversible cross-linking of peptide-bound glutamine and lysine residues^29^ and is widely expressed in the small intestine, brain, skin, and mucosa.^30^ Recent studies highlight its crucial role in both physiological and pathological processes. In the skin and mucosa, Tgm3 facilitates epidermal differentiation and keratinocyte envelope formation by cross-linking structural proteins such as involucrin, loricrin, and small proline-rich proteins.^31^ Moreover, Tgm3 functions as a key suppressor of EMT and PI3K/AKT signaling in CRC,^32^ highlighting its potential as a novel therapeutic target.

Gene Ontology (GO) enrichment analysis classified the differentially expressed genes (DEGs) into molecular function (MF), biological process (BP), and cellular component (CC) categories.^33^ In the B­[a]­P/DSS vs control comparison (Figure S3B), BP terms were predominant, with the extracellular matrix (ECM) ranking highest in the CC category. For PI vs B­[a]­P/DSS (Figure 4C), BP terms were significantly enriched, with response to wounding as the top-ranked term. In PII vs B­[a]­P/DSS, MF terms such as secondary active transmembrane transporter activity showed significant enrichment (Figure S4C). Lastly, PIII vs B­[a]­P/DSS exhibited a more balanced distribution of enriched MF and BP terms, though most lacked statistical significance (Figure S4D).

To elucidate functional networks, cnetplots were generated based on the top 30 GO terms. In the B­[a]­P/DSS vs Cont comparison (Figures S3C and S4E,F), significant alterations were observed in CC terms, particularly in actin-based cell projections and collagen-containing ECM. Tgm3 was notably downregulated within the collagen-related category, suggesting compromised ECM stability and increased tumor invasion potential. BP terms highlighted the regulation of inflammatory response and leukocyte migration, with increased expression of proinflammatory genes such as Ccl3 (chemokine [C–C motif] ligand 3) and Cxcl9 (C–X–C motif chemokine ligand 9), reflecting immune cell recruitment and heightened inflammation.^34^

In the PI vs B­[a]­P/DSS comparison (Figure 4D and Figure S5B,C), upregulated genes within CC terms such as collagen-containing ECM (e.g., Tgm3) indicate ECM repair and stabilization. The MF term secondary active transmembrane transporter activity, involving genes like Slc37a2, suggests restored nutrient transport and metabolic function. BP terms such as response to wounding (e.g., Tgm3 and IL-6) and epithelial tube morphogenesis (e.g., Wnt4 and Fgfr1 [fibroblast growth factor receptor 1]) suggest improved structural and functional restoration of the intestinal epithelium.^35,36^

PII vs B­[a]­P/DSS (Figure S5D–F) showed significant changes in CC terms, such as the brush border membrane and cell cortex region, indicating restored nutrient absorption and cytoskeletal organization. BP terms like organic anion transport and positive regulation of peptide secretion suggest enhanced secretion and metabolic detoxification, with genes such as *Slc37a2* playing key roles. Upregulated MF terms such as secondary active transmembrane transporter activity highlight metabolic and transport recovery under PIC treatment.

For PIII vs B­[a]­P/DSS (Figure S6A–C), CC terms such as the GABA-A receptor complex and Cul2-RING ubiquitin ligase complex indicate potential involvement in neural and protein degradation pathways, which may influence inflammatory resolution.^37,38^ BP terms related to endopeptidase activity (e.g., *Ctrb1*) and lipase activity (e.g., *Pla2g2a* [phospholipase A2 group IIA]) suggest metabolic activation and tissue repair.^39^ These findings collectively demonstrate the temporal and group-specific effects of PIC intervention, highlighting its potential to modulate inflammation, restore intestinal barrier function, and reshape the tumor microenvironment.

### 3.4 Differential Gene Expression Analysis in the B­[a]­P/DSS-Induced CRC Model Treated with PIC

DEGs were annotated within the Kyoto Encyclopedia of Genes and Genomes (KEGG) metabolic pathway database and visualized in a KEGG pathways dot plot, where dot size represents enrichment levels, allowing for an integrated display of multiple data points. Additionally, KEGG metabolic pathway maps were used to illustrate the spatial distribution of DEGs, with color coding indicating the extent of upregulation or downregulation.^40^

The KEGG pathway dot plot and metabolic pathway map results demonstrated that the most active pathways in the B­[a]­P/DSS group, compared to the control, were cytokine-cytokine receptor interaction and the PI3K/AKT signaling pathway (Figures S3D,E and S6F). Notably, the PI group exhibited significant differences compared to the B­[a]­P/DSS group in cytokine-cytokine receptor interaction­(Figure 4F) and the Wnt signaling pathway (Figure S6G). The PII group displayed the most notable activity in the KEGG pathway related to mineral absorption; however, this pathway did not achieve statistical significance in the dot plot (Figure S6D). Conversely, the PIII group showed statistically significant alterations in the pancreatic secretion pathway in the KEGG pathway dot plot (Figure S6E). These findings suggest that PIC may mitigate B­[a]­P/DSS-induced intestinal damage, supporting its potential role as a protective agent against CRC. The study highlights the therapeutic potential of PIC in developing novel strategies for CRC management.

### 3.5 Effects of PIC Intervention on the PI3K/AKT/mTOR Pathway and *Tgm3* Expression in B­[a]­P/DSS-Induced CRC Mice

The PI3K/AKT/mTOR signaling pathway regulates key cellular functions, including proliferation, survival, DNA repair, apoptosis, and gene transcription.^41^ To investigate the mechanism of PIC intervention further, we performed Western blot analysis based on RNA expression findings in colon tissues.

Figure 5 illustrates the effects of different PIC intervention time points on PI3K/AKT/mTOR pathway activation and Tgm3 expression in the B­[a]­P/DSS-induced CRC mouse model. Representative Western blot images (Figure 5A,B) and corresponding quantitative analyses (Figure 5C–F) demonstrate the modulation of pathway components and Tgm3 levels. Quantitative analysis of p-PI3K/PI3K (Figure 5C) showed that the PI group showed significantly lower expression compared with the B­[a]­P/DSS-induced group. The expression of p-AKT/AKT was low in the PI group (Figure 5D). Further analysis of p-mTOR/mTOR levels (Figure 5E) showed a significant reduction in the PI group relative to the induced group. Additionally, the expression of Tgm3 in the PI group (Figure 5F) was significantly upregulated compared with that in the induced group. These findings suggest that early intervention targeting the PI3K/AKT/mTOR pathway may mitigate inflammation and facilitate tissue repair in CRC.

**Figure 5:**
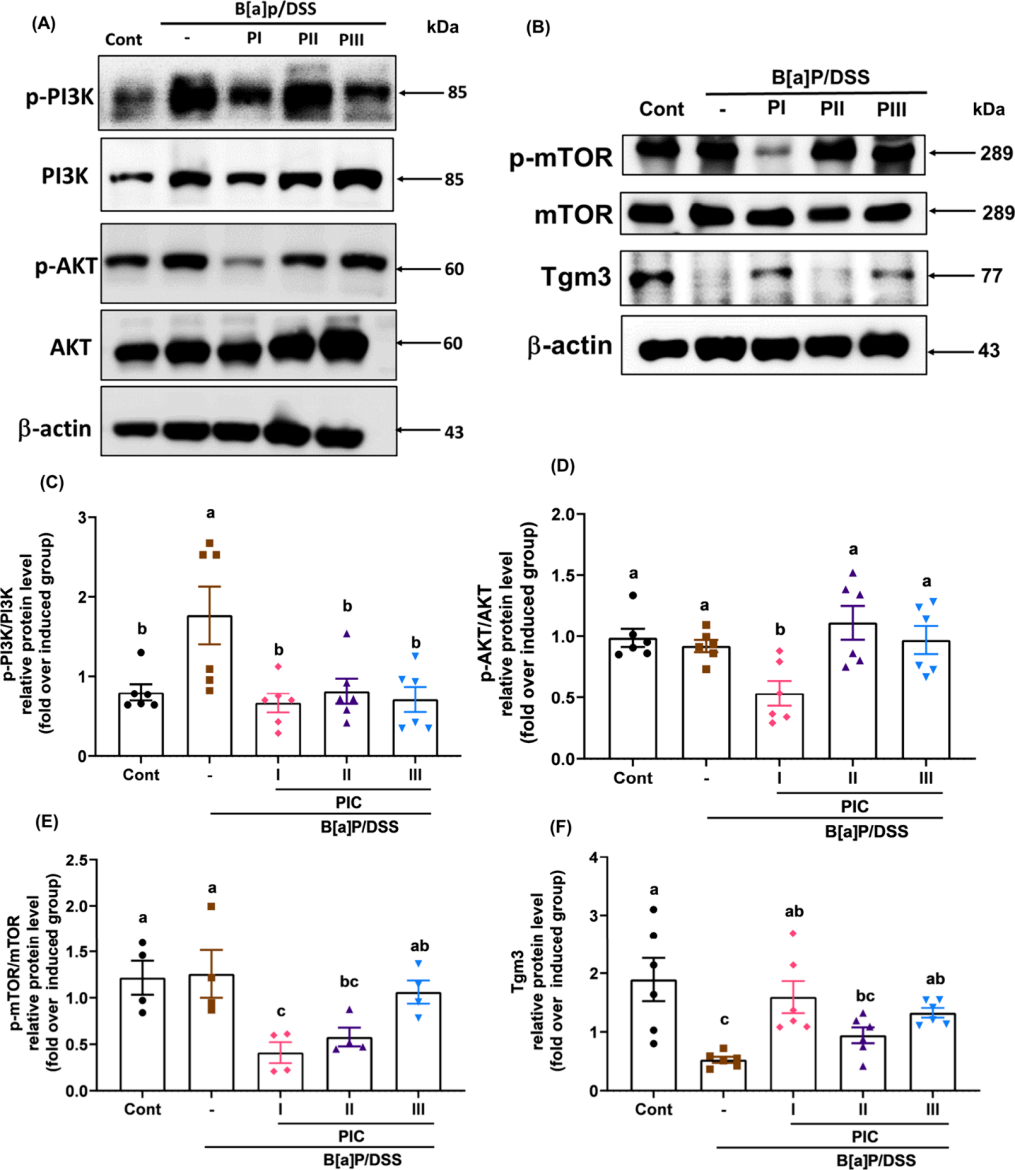
Effects of PIC on the PI3K/AKT/mTOR signaling pathway and Tgm3 expression in the colonic tissues of B­[a]­P/DSS-induced colorectal cancer mice. (A) Representative Western blot images of p-PI3K, PI3K, p-AKT, AKT, and β-actin. (B) Representative Western blot images of p-mTOR, mTOR, Tgm3, and β-actin. (C–F) Quantification of protein expression levels, including (C) p-PI3K/PI3K, (D) p-AKT/AKT, (E) p-mTOR/mTOR, and (F) Tgm3, analyzed using ImageJ. Data are presented as means ± SEM (*n* = 4–6 per group). Statistical significance was determined by one-way ANOVA followed by Duncan’s multiple range test. Different letters (a–c) indicate significant differences among groups (*p* < 0.05).

### 3.6 Effects of PIC Intervention at Different Time Points on Gut Microbiota in B­[a]­P/DSS-Induced CRC Mice

Partial least-squares discriminant analysis (PLS-DA; Figure 6A) revealed distinct microbial community compositions among the experimental groups. The B­[a]­P/DSS group exhibited the greatest deviation from the control group, reflecting severe dysbiosis associated with inflammation and tumorigenesis. Notably, the PI group clustered more closely with the control group, suggesting that full-course PIC intervention partially restored microbial homeostasis. UpSet plot analysis (Figure S7A) further illustrated shared and unique operational taxonomic units (OTUs) across groups, with the PIII group displaying the highest species count among the PIC-treated groups, indicative of partial microbial diversity recovery with delayed intervention.

**Figure 6:**
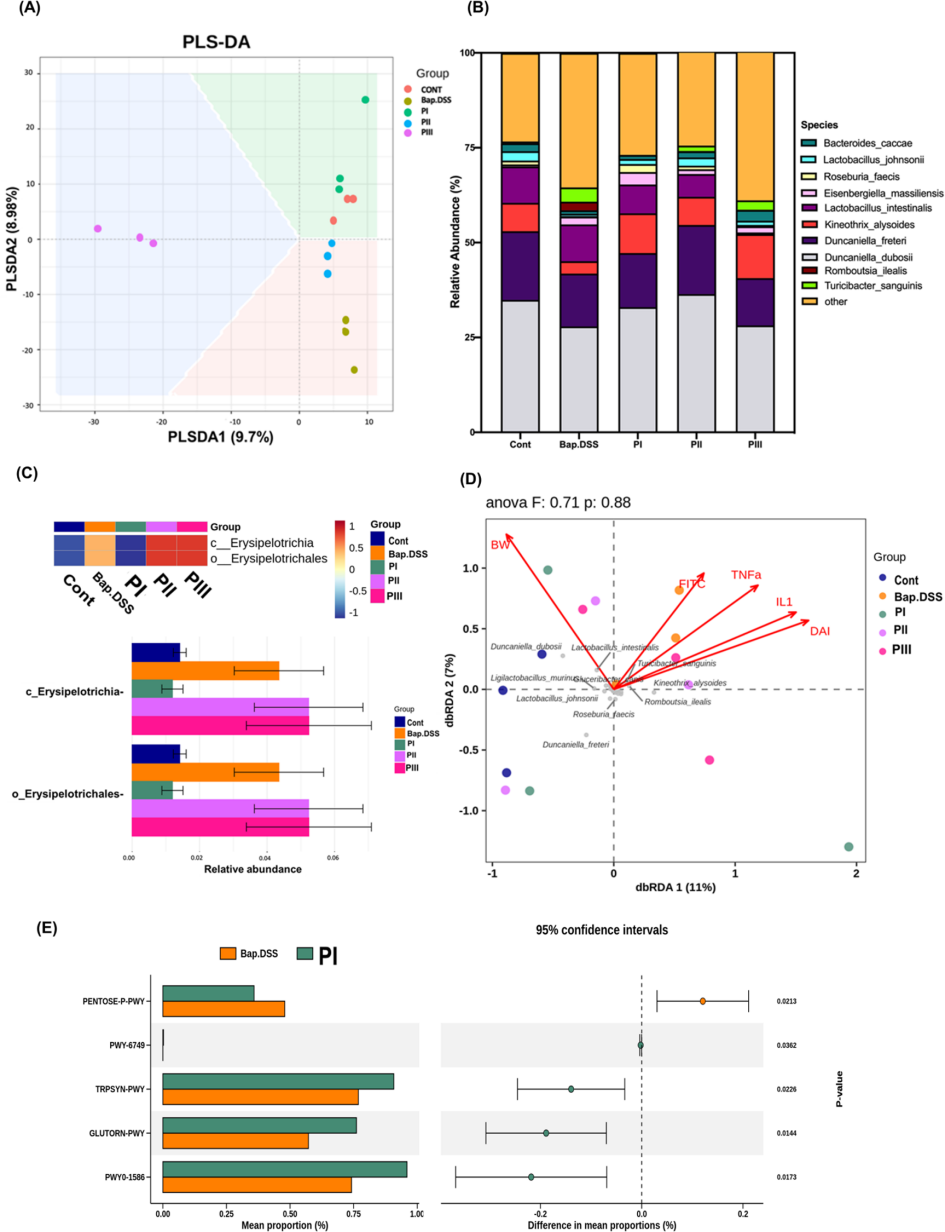
PIC altered gut microbiota composition in B­[a]­P/DSS-induced colorectal cancer mice. (A) Partial least-squares discriminant analysis (PLS-DA). (B) Relative abundance of the top 10 species of the gut microbiota among the five groups. (C) Biomarker group heat map and relative abundance bar plot in LDA_4.0. (D) Distance-based redundancy analysis (db-RDA). The db-RDA plot shows correlations between gut microbiota composition and final BW, FITC, TNF-α, IL-1β, and DAI. (E) PICRUSt (Phylogenetic Investigation of Communities by Reconstruction of Unobserved States) pathway and dot-and-whisker plot in B­[a]p and PI groups. *n* = 3 per group. A *p* value <0.05 indicated a significant difference.

Analysis of the relative abundance of the top 10 gut microbial species (Figure 6B) demonstrated significant alterations in microbial community structure across the groups. Dysbiosis in the B­[a]­P/DSS group was characterized by an overrepresentation of pathogenic species, including *Turicibacter sanguinis* and *Romboutsia ilealis*.^42,43^ In contrast, PIC intervention, particularly in the PI group, promoted enrichment of beneficial butyrate-producing species such as *Roseburia faecis* and *Kineothrix alysoides*.^44,45^ While *K. alysoides* was most abundant in the PIII group, followed by the control and PI groups, *R. faecis* showed the highest relative abundance in the PI group, underscoring the therapeutic potential of early PIC intervention.

Species-specific analysis (Figure S7B–E) provided further insights into these microbial alterations. *T. sanguinis*, a proinflammatory bacterium associated with gut dysbiosis, was significantly elevated in the B­[a]­P/DSS group. PIC intervention, particularly in the PI group, led to the most pronounced reduction (*p* < 0.05), while the PII and PIII groups exhibited intermediate decreases. Similarly, *R. ilealis*, which is associated with neurodevelopmental disorders and metabolic diseases, was markedly enriched in the B­[a]­P/DSS group but was reduced to near-control levels following PIC treatment, with the PI group showing the greatest effect.

The heat map and relative abundance bar plot (Figure 6C) further illustrate changes in biomarker taxa, particularly *Erysipelotrichia*, a bacterial class strongly associated with dysbiosis and inflammation.^46^ Notably, the PI group exhibited a substantial reduction in *Erysipelotrichia* abundance, while the PII and PIII groups retained higher levels, indicating reduced efficacy of delayed interventions in suppressing this opportunistic taxon. Canonical correspondence analysis (Figure 6D) identified the most significant environmental drivers of microbial community shifts. The control and PI groups were negatively correlated with inflammatory markers and intestinal damage. Conversely, the B­[a]­P/DSS group was positively correlated with markers of intestinal ulceration, underscoring the protective effects of early PIC intervention.

Predicted functional pathway analysis (Figure 6E and Figure S2F,G) provided further insights into microbial metabolic activity. The B­[a]­P/DSS group exhibited enrichment in pathways linked to oxidative stress and tumor progression, such as the nonoxidative pentose phosphate pathway (NONOXIPENT-PWY) and NAD biosynthesis (NAD-BIOSYNTHESIS-II).^47^ In contrast, the PI group demonstrated significant upregulation of pathways involved in butyrate synthesis (GLUTORN-PWY) and tryptophan biosynthesis (TRPSYN-PWY), aligning with the observed enrichment of *R. faecis* and *K. alysoides*.^48,49^ These findings suggest that PIC intervention promotes the production of SCFAs and anti-inflammatory metabolites, which are essential for maintaining intestinal barrier integrity and modulating immune responses. While the PIII group exhibited partial recovery, its metabolic pathway activity remained intermediate, highlighting the reduced efficacy of delayed intervention.

Analysis of SCFA concentrations (Figure S7F–I) further highlighted the functional impact of microbial shifts. The PI group demonstrated the highest levels of acetic acid and propionic acid, metabolites critical for maintaining intestinal barrier integrity and energy homeostasis. Additionally, increased butyrate levels in the control and PIII groups emphasized the role of SCFAs in preventing tumor progression and promoting intestinal health. Collectively, these findings underscore the critical role of early and sustained PIC intervention in modulating gut microbiota composition and function, mitigating dysbiosis, and supporting intestinal homeostasis in B­[a]­P/DSS-induced CRC.

## 4 Discussion

B­[a]­P, a highly carcinogenic polycyclic aromatic hydrocarbon (PAH), induces DNA adduct formation, leading to mutations and promoting CRC development.^50^ When combined with DSS, a widely used inducer of colonic inflammation, the B­[a]­P/DSS model effectively recapitulates the interplay between chronic inflammation and PAH exposure in CRC progression.^51^ This model provides a valuable platform for investigating the complex interactions between environmental carcinogens, inflammation, and tumorigenesis, facilitating the development of targeted therapeutic strategies.^17^ This study highlights the chemopreventive potential of PIC in a B­[a]­P/DSS-induced CRC model, emphasizing the importance of early intervention. Our findings suggest that PIC mitigates tumor progression by modulating inflammatory responses, restoring gut microbiota homeostasis, and suppressing the PI3K/AKT/mTOR signaling pathway, accompanied by upregulation of Tgm3.

A key finding of this study is the timing-dependent efficacy of PIC administration. Early and sustained intervention (PI group) resulted in the most significant tumor suppression, inflammatory modulation, and microbiota restoration, whereas delayed interventions (PII and PIII groups) exhibited progressively weaker effects. The PI group demonstrated notable reductions in tumor burden, DAI, and intestinal permeability, along with enhanced expression of *Tgm3* and suppression of the PI3K/AKT/mTOR pathway. In contrast, the PIII group exhibited only partial recovery, with moderate reductions in tumor burden and inflammatory markers, suggesting that late intervention may be insufficient to reverse established tumor-promoting processes. The PII group, representing an intermediate intervention strategy, showed moderate improvements, reinforcing the notion that timing plays a critical role in maximizing the therapeutic effects of PIC. These findings underscore the importance of early intervention in preventing tumor progression, likely due to its ability to suppress early inflammatory responses and restore gut homeostasis before irreversible tumorigenic changes occur. This aligns with previous studies indicating that early modulation of oncogenic pathways enhances therapeutic efficacy.^52^

The PI3K/AKT/mTOR pathway is a key regulator of CRC progression, cell proliferation, and survival, frequently hyperactivated in CRC.^53,54^ Since mTOR functions as a key downstream effector in this signaling cascade, we included it in our mechanistic analysis to comprehensively assess the modulatory effects of PIC along the entire PI3K/AKT/mTOR axis. Western blot analysis confirmed that PIC significantly reduced phosphorylated PI3K, AKT, and mTOR levels, particularly in the PI group, indicating that early intervention more effectively disrupts oncogenic signaling. Importantly, PIC treatment led to *Tgm3* upregulation, which was correlated with suppression of the PI3K/AKT/mTOR signaling pathway. Given the established role of *Tgm3* in epithelial repair and ECM stability, as well as its known function in inhibiting EMT,^32^ *Tgm3* may contribute to the attenuation of oncogenic signaling observed in this study. Nevertheless, we fully acknowledge that the precise causal relationship between *Tgm3* upregulation and PI3K/AKT/mTOR pathway suppression remains to be further validated. While our current findings support the hypothesis that PIC may modulate this oncogenic pathway in part through *Tgm3* regulation, additional mechanistic studies using *Tgm3* knockdown or overexpression models will be necessary to confirm this association. In addition, although other pathways such as Wnt signaling and TNF-related inflammatory cascades were also enriched in our transcriptomic analysis, our mechanistic validation primarily focused on the PI3K/AKT/mTOR axis due to its central role in CRC pathogenesis and its documented regulation by *Tgm3*. Notably, several enriched pathways identified in the KEGG analysis (e.g., cytokine–cytokine receptor interaction and Wnt signaling) are functionally interconnected with the PI3K/AKT signaling cascade, suggesting that PIC may exert broader regulatory effects across multiple oncogenic networks. However, to maintain a focused mechanistic narrative, we prioritized experimental validation of the PI3K/AKT/mTOR pathway, which was further supported by previous literature linking PIC to this signaling axis. Future studies may expand on this work by investigating the interplay between PIC, gut microbiota, and additional pathways such as Wnt and TNF signaling.

*Slc37a2* was upregulated in the PI and PII groups; as an endoplasmic reticulum glucose-6-phosphate/phosphate exchanger, *Slc37a2* restrains macrophage glycolysis and dampens proinflammatory activation, suggesting an immunometabolic link to barrier support in inflamed colon tissues.^28^ By contrast, *Ctrb1* enrichment in the PIII group likely reflects activation of an LRH-1 (NR5A2)-driven epithelial program: human LRH-1 preserves epithelial integrity, mitigates T cell-mediated colitis, and binds the proximal *Ctrb1* promoter, making *Ctrb1* a sensitive marker of LRH-1 pathway engagement rather than a proven effector.^55^ Targeted perturbation will be required to establish causality. These observations warrant future mechanistic studies to define the causal roles of *Slc37a2* and *Ctrb1* in CRC pathogenesis and their potential for therapeutic modulation.

Dysbiosis is increasingly recognized as a significant driver of CRC development.^56^ B­[a]­P/DSS exposure induced microbiota imbalance, characterized by an overrepresentation of pathogenic species (*T. sanguinis* and *R. ilealis*) and a reduction in beneficial butyrate-producing bacteria. PIC treatment, particularly in the PI group, restored microbial diversity, increasing *R. faecis* and *K. alysoides*, species known for their SCFA production and immunomodulatory properties.^44,49^ While PI intervention resulted in the most significant microbiota restoration, the PIII group exhibited only partial recovery, showing moderate reductions in proinflammatory taxa. These findings suggest that delayed PIC intervention may be insufficient for fully restoring microbiota homeostasis. Although beneficial species such as *K. alysoides* exhibited a modest recovery, RNA-seq data revealed only partial suppression of inflammatory pathways and weaker effects on repair-related genes compared to the PI group, further highlighting the importance of early intervention. Although the observed microbial shifts coincided with reduced inflammation and tumorigenesis, causal relationships remain to be established. Future studies employing fecal microbiota transplantation (FMT) or gnotobiotic mouse models will help determine whether specific taxa such as *R. faecis* or *T. sanguinis* directly mediate the protective effects of PIC.

Functional pathway predictions further supported these microbiota–host interactions. The B­[a]­P/DSS group exhibited enrichment in tumor-promoting pathways, such as the NONOXIPENT-PWY and NAD biosynthesis, both associated with oxidative stress and ECM remodeling.^47^ In contrast, the PI group exhibited significant enrichment in SCFA production and metabolic recovery pathways, correlating with reduced inflammation and enhanced tissue repair. The increased levels of SCFAs, particularly butyrate, acetate, and propionate, in the PI group likely contributed to the suppression of proinflammatory signals and the promotion of epithelial barrier integrity, as further supported by RNA-seq data. Mechanistically, distinct SCFAs likely act on epithelial and immune compartments to converge on PI3K/AKT/mTOR-related signaling.^57^ For example, acetate can down-modulate PI3K/AKT in colorectal cancer cells, reducing the immune-checkpoint ligand PVR/CD155 and thereby enhancing CD8^+^ T cell effector responses in the tumor microenvironment.^58^ Propionate has been shown to upregulate epithelial stress ligands (MICA/B) and engage an mTORC2/PDK1/AKT–p21 axis, indicating context- and cell-type-dependent signaling.^59^ Butyrate, beyond its barrier-supporting and anti-inflammatory actions, can inhibit AKT/ERK and dampen mTOR/S6K via HDAC-dependent mechanisms in CRC cells.^60^ Collectively, these data support a model in which acetate, propionate, and butyrate exert cell-type-specific effects that intersect with PI3K/AKT/mTOR, complementing the PIC-driven suppression observed in our study.

Previous studies have shown that PIC suppresses DSS-induced colonic inflammation by modulating NF-κB, STAT3, and epidermal growth factor receptor (EGFR/ERK) signaling.^61^ Additionally, PIC has been reported to inhibit azoxymethane (AOM)/DSS-induced colon tumor growth by downregulating cyclooxygenase-2 (COX-2) and reducing monocyte chemotactic protein 1 (MCP-1) and programmed death-ligand 1 (PD-L1) expression.^62^ However, this study is the first to evaluate the timing-dependent effects of PIC on transcriptomic changes in intestinal tissues and gut microbiota in a B­[a]­P/DSS-induced CRC model. Unlike previous studies that primarily focused on inflammatory modulation, our findings provide novel insights into the host–microbiota interplay in CRC prevention. Stilbenes exhibit broad anti-CRC actions by modulating inflammation, oxidative stress, proliferation, apoptosis, and metastasis. RES is extensively documented to inactivate PI3K/AKT signaling in CRC cells (e.g., via BMP7 upregulation) and to modulate gut barrier function and the microbiota in colitis models.^63,64^ Pterostilbene shows a pharmacokinetic advantage with higher oral bioavailability than RES.^65^ In contrast, PIC in our study converges on oncogenic signaling (PI3K/AKT/mTOR) and concurrently reinforces epithelial barrier integrity and reshapes gut microbiota, supporting a dual host–microbiota mechanism particularly relevant to inflammation-driven CRC.

Despite these promising findings, several limitations must be acknowledged. First, this study primarily relied on RNA-seq data to assess gene expression changes, and although we validated key targets such as PI3K, AKT, and mTOR at the protein level, the proposed association between Tgm3 upregulation and suppression of the PI3K/AKT/mTOR pathway remains correlative. Future mechanistic studies using *Tgm3* knockout or overexpression models will be essential to confirm whether *Tgm3* directly mediates the oncogenic suppression observed in response to PIC intervention. Second, while gut microbiota profiling revealed substantial restoration of microbial diversity, particularly enrichment of beneficial SCFA-producing taxa such as *Roseburia faecis* and *Kineothrix alysoides*, our current data do not establish a direct causal relationship between these microbial changes and the chemopreventive effects of PIC. Future work utilizing FMT or gnotobiotic mouse models may clarify the functional contributions of specific taxa to CRC attenuation. Third, although this study integrated transcriptomic and microbial profiling, we did not perform advanced network-based analyses (e.g., host–microbiota coregulation modeling or multiomics network inference). We recognize that these system-level approaches will be valuable in future studies to more precisely elucidate how PIC shapes host–microbiota interactions and downstream signaling cascades. Additionally, while the B­[a]­P/DSS-induced CRC model effectively simulates inflammation-associated tumorigenesis, it may not fully reflect the chronic, heterogeneous nature of human CRC. Limitations include synchronized inflammation onset, limited adaptive immunity, and constrained microbiota diversity. Complementary models, such as genetically engineered mice or patient-derived organoids, are warranted to validate and expand the translational relevance of our findings.

In conclusion, this study demonstrates that PIC intervention effectively suppresses CRC progression by modulating inflammation, restoring gut microbiota homeostasis, and inhibiting the PI3K/AKT/mTOR pathway, accompanied by Tgm3 upregulation. Early and sustained intervention (PI group) provided the most robust chemopreventive effects, while delayed interventions (PII and PIII) demonstrated progressively weaker outcomes. The dual modulation of gut microbiota and host transcriptomic responses positions PIC as a promising candidate for CRC prevention and therapeutic intervention. Although we quantified total SCFAs, we did not resolve SCFA subtype- and cell-type-specific signaling *in vivo*. Targeted metabolomics (acetate/propionate/butyrate), receptor profiling (e.g., FFAR2), and cell-resolved assays in epithelial and immune subsets (e.g., CD8^+^ T cells) will be required to establish causal links to PI3K/AKT/mTOR modulation. Moving forward, translational studies should validate these findings in clinical settings and further dissect the mechanistic links between microbial metabolites, host signaling pathways, and gene expression programs such as *Tgm3*, ultimately paving the way for microbiota-targeted CRC therapies.

## Supplementary Material



## Notes

Body weight, food and water intake, representative photographs of organs, liver weight, AST, tumor incidence, histological scoring systems, transcriptomic analysis (RNA-seq), and gut microbiota profiling in the B­[a]­P/DSS-induced colorectal cancer mouse model (PDF)

## Abbreviations

AhR: aryl hydrocarbon receptor
AKT: protein kinase B
ALT: alanine aminotransferase
AOM: azoxymethane
AST: aspartate aminotransferase
B­[a]­P: benzo­[*a*]­pyrene
BP: biological process
BUN: blood urea nitrogen
CC: cellular component
CCL3: chemokine (C–C motif) ligand 3
COX-2: cyclooxygenase-2
CRC: colorectal cancer
CREA: creatinine
ctrb1: chymotrypsinogen B1
Cxcl9: C–X–C motif chemokine ligand 9
DAI: disease activity index
DEGs: differentially expressed genes
DSS: dextran sulfate sodium
ECM: extracellular matrix
ERK: epidermal growth factor receptor
Fgfr1: fibroblast growth factor receptor 1
FITC: fluorescein isothiocyanate
FMT: fecal microbiota transplantation
GC-MS: gas chromatography–mass spectrometry
GO: gene ontology
H&E: hematoxylin and eosin
HIF-1: hypoxia-inducible factor-1
IL: interleukin
KEGG: Kyoto Encyclopedia of Genes and Genomes
MCP-1: monocyte chemotactic protein 1
MF: molecular function
MMPs: matrix metalloproteinases
mTOR: mammalian target of rapamycin
NAD-BIOSYNTHESIS-II: NAD biosynthesis
NF-κB: nuclear factor kappa B
NGS: next-generation sequencing
NONOXIPENT-PWY: nonoxidative pentose phosphate pathway
Nrf2: nuclear factor erythroid 2-related factor 2
OTUs: operational taxonomic units
PAHs: polycyclic aromatic hydrocarbons
PD-L1: programmed death-ligand 1
PI3K: phosphoinositide 3-kinase
PIC: piceatannol
Pla2g2a: phospholipase A2 group IIA
PLS-DA: partial least-squares discriminant analysis
RES: resveratrol
ROS: reactive oxygen species
SCFAs: short-chain fatty acids
SLC37A2: solute carrier family 37 member 2
TC: total cholesterol
TG: triacylglycerol
Tgm3: transglutaminase 3
TME: tumor microenvironment
TNF-α: tumor necrosis factor-α
