# Supplementary material for: Piceatannol Attenuates Benzo[a]pyrene/DSS-Induced Colorectal Cancer in Mice via Modulation of Gut Microbiota and Inhibition of the PI3K/AKT/mTOR Pathway
Source: J Agric Food Chem. 2025 Sep 25;73(40):25379–93. doi: 10.1021/acs.jafc.5c05807 (PMC12512187; doi:10.1021/acs.jafc.5c05807)
Supplement: Supplementary file 1 [file jf5c05807_si_001.pdf]

Piceatannol Attenuates Benzo[a]pyrene/DSS-Induced Colorectal Cancer in Mice via Modulation of Gut Microbiota and Inhibition of the PI3K/AKT/mTOR Pathway

Pin-Yu Ho[a], Yen-Chun Koh[a], Wei-Sheng Lin[a, b], Chin-Jui Ho[c, d], Anju Majeed[e], Chi-Tang Ho[f], and Min-Hsiung Pan[a, g, h]*

[a] Institute of Food Science and Technology, National Taiwan University, Taipei 10617, Taiwan

[b] Department of Food Science, National Quemoy University, Quemoy County 89250, Taiwan

[c] School of Pharmacy, College of Medicine, National Taiwan University, Taipei 100025, Taiwan

[d] Program in Neuroscience, Baylor College of Medicine, Houston, TX 77030, USA

[e] Sami-Sabinsa Group Limited, Bengaluru 560058, Karnataka, India

[f] Department of Food Science, Rutgers University, New Brunswick, New Jersey 08901-8520, USA

[g] Department of Public Health, China Medical University, Taichung 40402, Taiwan

[h] Department of Health and Nutrition Biotechnology, Asia University, 413305 Taichung City, Taiwan

*** Correspondence:**
Dr. Min-Hsiung Pan
Institute of Food Science and Technology,
National Taiwan University,
No. 1, Section 4, Roosevelt Road, Taipei 10617, Taiwan.
Tel. no. +886-2-33664133

Fax. no. +886-2-33661771
E-mail: mhpan@ntu.edu.tw

Supplementary Table 1. Effect of PIC on initial body weight, final body weight, body weight gain, food intake, and water intake in B[a]P/DSS-induced colorectal cancer mice.

Groups	Initial body weight (g)	Final body weight (g)	Body weight gain (g)	Food intake (g/day)	Water intake (mL/day)
Cont	29.66 ± 0.36[a]	44.52 ± 0.45[a]	14.86 ± 0.41[a]	5.98 ± 0.20[a]	4.17 ± 0.27[a]
B[a]P/DSS	28.86 ± 0.42[a]	42.11 ± 0.46[b]	13.25 ± 0.65[a]	6.19 ± 0.64[a]	4.17 ± 0.30[a]
PI	29.01 ± 0.29[a]	44.04 ± 0.81[a]	15.03 ± 0.82[a]	5.76 ± 0.35[a]	4.47 ± 0.39[a]
PII	29.06 ± 0.3[a]	43.51 ± 0.61[ab]	14.45 ± 0.68[a]	6.13 ± 0.58[a]	4.63 ± 0.31[a]
PIII	28.97 ± 0.29[a]	43.12 ± 0.72[ab]	14.15 ± 0.65[a]	5.63 ± 0.44[a]	4.59 ± 0.38[a]

Data are expressed as mean ± SEM. Food and water intake values represent the average across the entire 18-week experimental period. Statistical significance among the five groups was analyzed by one-way ANOVA followed by Duncan's multiple range test. Values with different superscript letters (a–b) indicate significant differences ($p < 0.05$).

Supplementary Table 2. Detailed scoring system for the disease activity index score used in study.

Score	Weight loss	Stool consistency	Fecal bleeding
0	<1%	Normal	Normal
1	1–5%	Slightly loose feces	Slightly bloody
2	6–10%	Loose	Bloody
3	11–15%	Very loose and wet	Blood in whole colon
4	>15%	Diarrhea	Blood in whole colon

Supplementary Table 3. Histological scoring system for colitis.

Histological changes		Severity of inflammation		Granulation tissue formation, mucosa	Hyperplasia, mucosa	Loss of goblet cells, mucosa
Score	0	None/normal mucosa-associated lymphoid tissue		None	Normal	Normal
	1	Minimal, multifocal infiltration of mononuclear cells (lymphocytes and macrophages) and neutrophils in the mucosal and submucosal layer	(≤25%)	Minimal, multifocal (≤25%)	Minimal (≤25%)	Minimal decrease of mucus (≤25%)
	2	Slight, multifocal infiltration of mononuclear cells (lymphocytes and macrophages) and neutrophils in the mucosal and submucosal layer	(26-50%)	Slight, multifocal (26-50%)	Slight (26-50%)	Slight decrease of mucus (26-50%)
	3	Moderate, diffuse infiltration of mononuclear cells (lymphocytes and macrophages) and neutrophils in the mucosal and submucosal layers	(51-75%)	Moderate, multifocal (51-75%)	Moderate (51-75%)	Moderate decrease of mucus (51-75%)
	4	Severe, diffuse infiltration of mononuclear cells (lymphocytes and macrophages) and neutrophils in the mucosal and submucosal layers	(76-100%)	Severe, diffuse (76-100%)	Severe (76-100%)	Severe depletion of mucus (76-100%)

Supplementary Table 4. Pathology - histologic assessment of colon tumors in mice.

group/number				Histopathological findings
Colon	Pathological number	Cont	1	-
			2	-
			3	-
		B[a]P/DSS	1	2 preneoplastic lesions; 5 adenomas
			2	2 preneoplastic lesions; 3 adenomas
			3	1 preneoplastic lesion; 2 adenomas
		PI	1	-
			2	-
			3	-
		PII	1	3 adenomas
			2	-
			3	-
		PIII	1	-
			2	-
			3	1 adenoma

-: No tumor lesions.

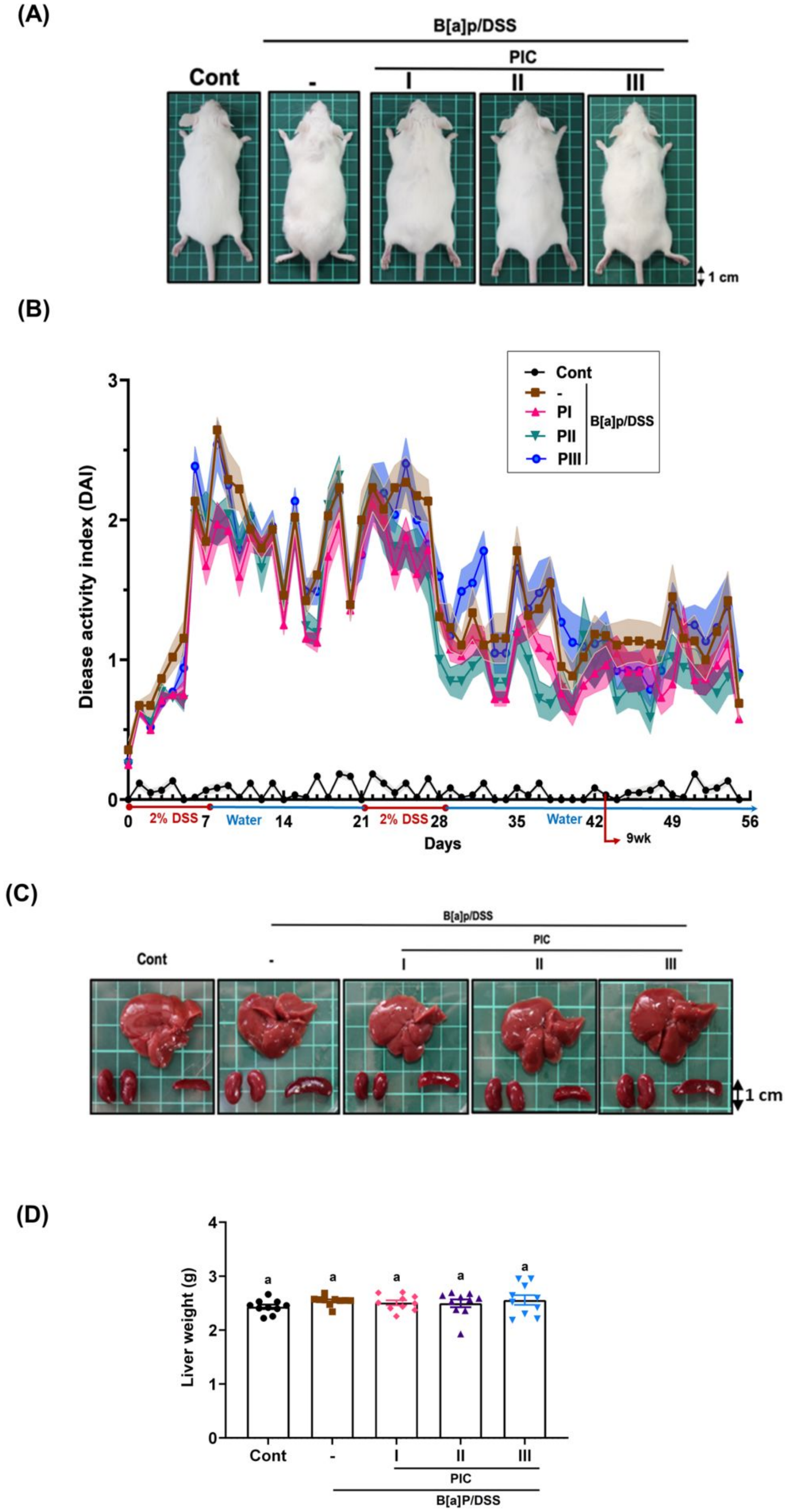

Supplementary Figure 1. Effect of PIC on the mouse appearance, DAI, and

organs in B[a]P/DSS-induced colorectal cancer mice.

(A) Representative photographs of each group mice after sacrifice. (B) Disease activity index (DAI). (C) Representative photographs of organs in each group (liver, kidneys, and spleen). (D) Liver weight. Data are presented as mean ± SEM; n=9–10 per group. p-values were determined by one-way ANOVA with subsequent Duncan’s multiple comparison test. The values with different letters (a, b, c) are significantly different ($p < 0.05$) between each group.

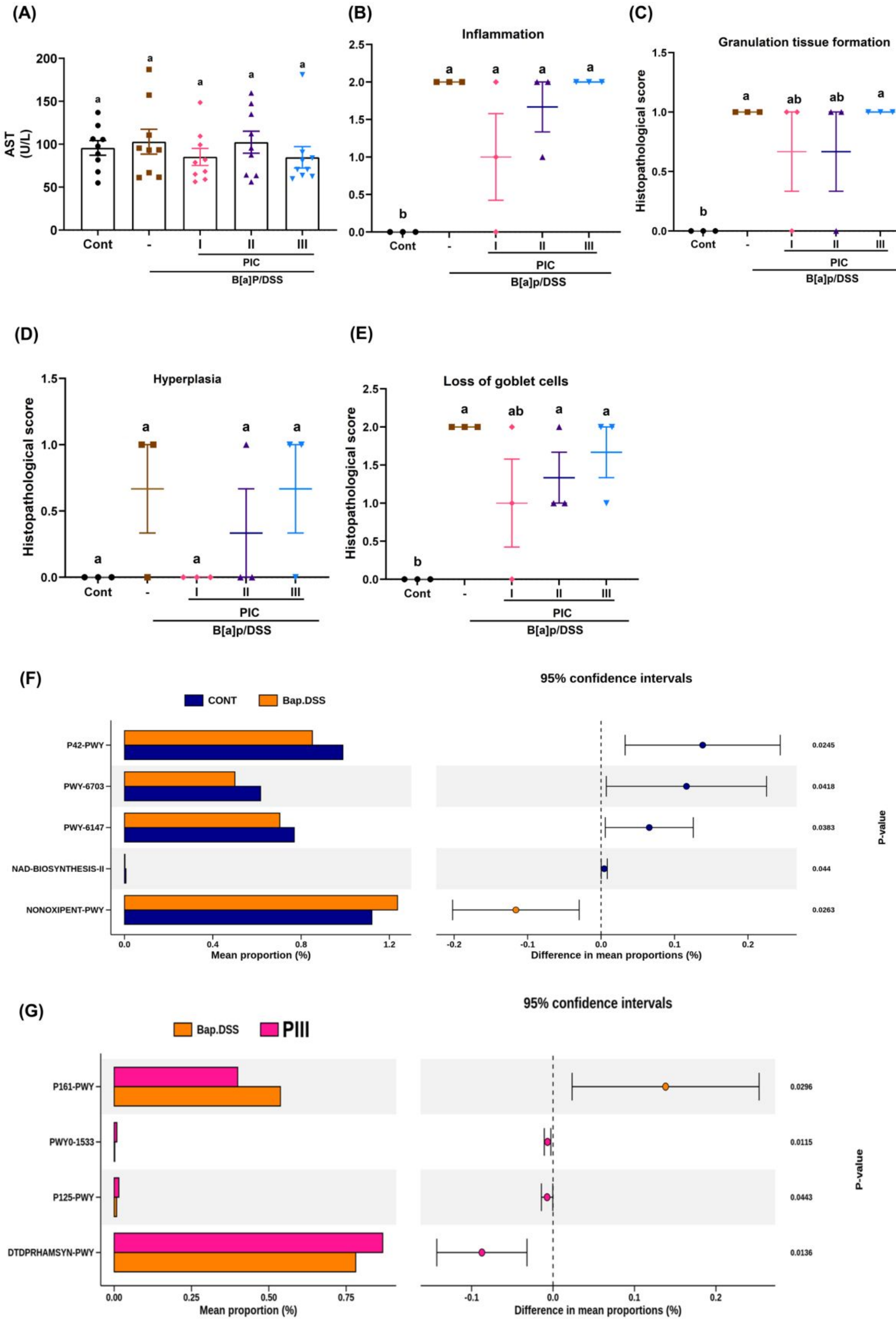

Supplementary Figure 2. Effect of PIC on AST, colon tumorigenesis, and PICRUSt pathway and dot-and-whisker plot in gut microbiota in B[a]P/DSS-induced colorectal cancer mice.

(A) AST. The histopathological score of (B) inflammation, (C) granulation tissue formation, (D) hyperplasia and (E) loss of goblet cells. (F) PICRUSt pathway and dot-and-whisker plot in control and PI groups. (G) PICRUSt pathway and dot-and-whisker plot in Bap.dss and PIII groups. n=3 per group. p value < 0.05 indicated significant difference.

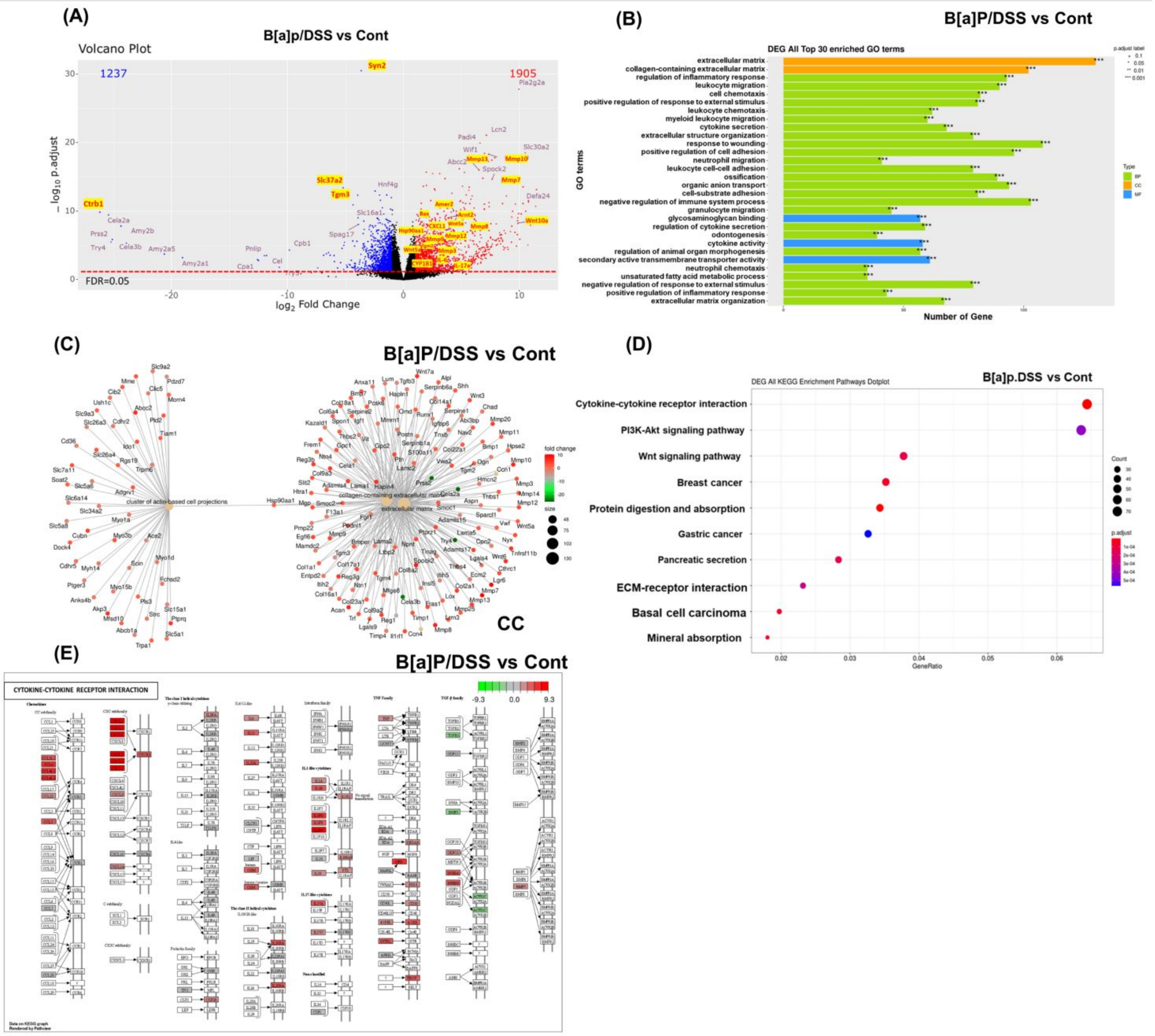

Supplementary Figure 3. RNA-seq analysis and pathway enrichment profiling of PIC intervention in B[a]P/DSS-induced colorectal cancer mice.

(A) Volcano plot of RNA-sequencing data of genes between B[a]p/DSS group vs Control group. Downregulated and upregulated genes are marked in blue and red, respectively, if they passed the threshold of false discovery rate (FDR)<0.05 and |log2FC|>0.5. (B) Top 30 Gene Ontology (GO) enrichment analysis in B[a]P/DSS vs Control group. (C) GO gene-concept network of core

genes between B[a]p/DSS group vs Control group in Cellular Component (CC). (D) KEGG pathways dotplot between B[a]p/DSS group and Control group. (E) Cytokine-cytokine receptor interactions in the KEGG pathway view of the gene map between B[a]p/DSS group and Control group. KEGG, Kyoto encyclopedia of genes and genomes. The enriched genes will be marked on the reaction pathway diagram, with red representing positive regulation and green representing negative regulation. *n*=3 per group.

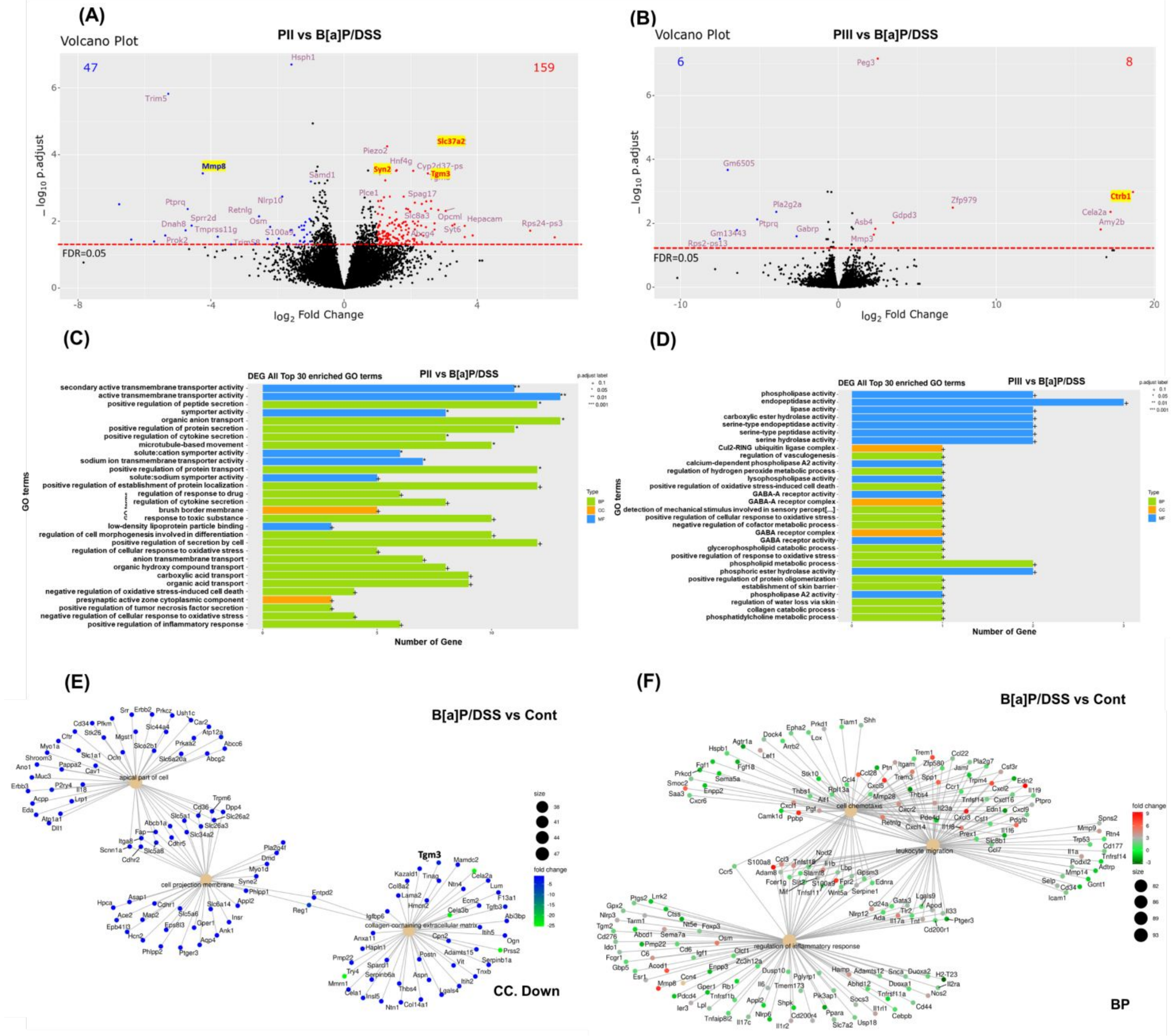

Supplementary Figure 4. RNA-seq analysis of the effect of PIC on transcriptional responses in B[a]P/DSS-induced colorectal cancer mice. (A) Volcano plot of RNA-sequencing data of genes between PII group vs B[a]P/DSS group. (B) Volcano plot of RNA-sequencing data of genes between PIII group vs B[a]P/DSS group. Downregulated and upregulated genes are marked in blue and red, respectively, if they passed the threshold of false discovery rate (FDR)<0.05 and |log2FC|>0.5. (C) Top 30 GO enrichment analysis in PII vs B[a]P/DSS group. (D) Top 30 GO enrichment analysis in PIII vs B[a]P/DSS group. (E) GO gene-concept network of core genes between

B[a]P/DSS group vs Control group in CC down regular. (F) GO gene-concept network of core genes between B[a]P/DSS group vs Control group in Biological Process (BP). n=3 per group.

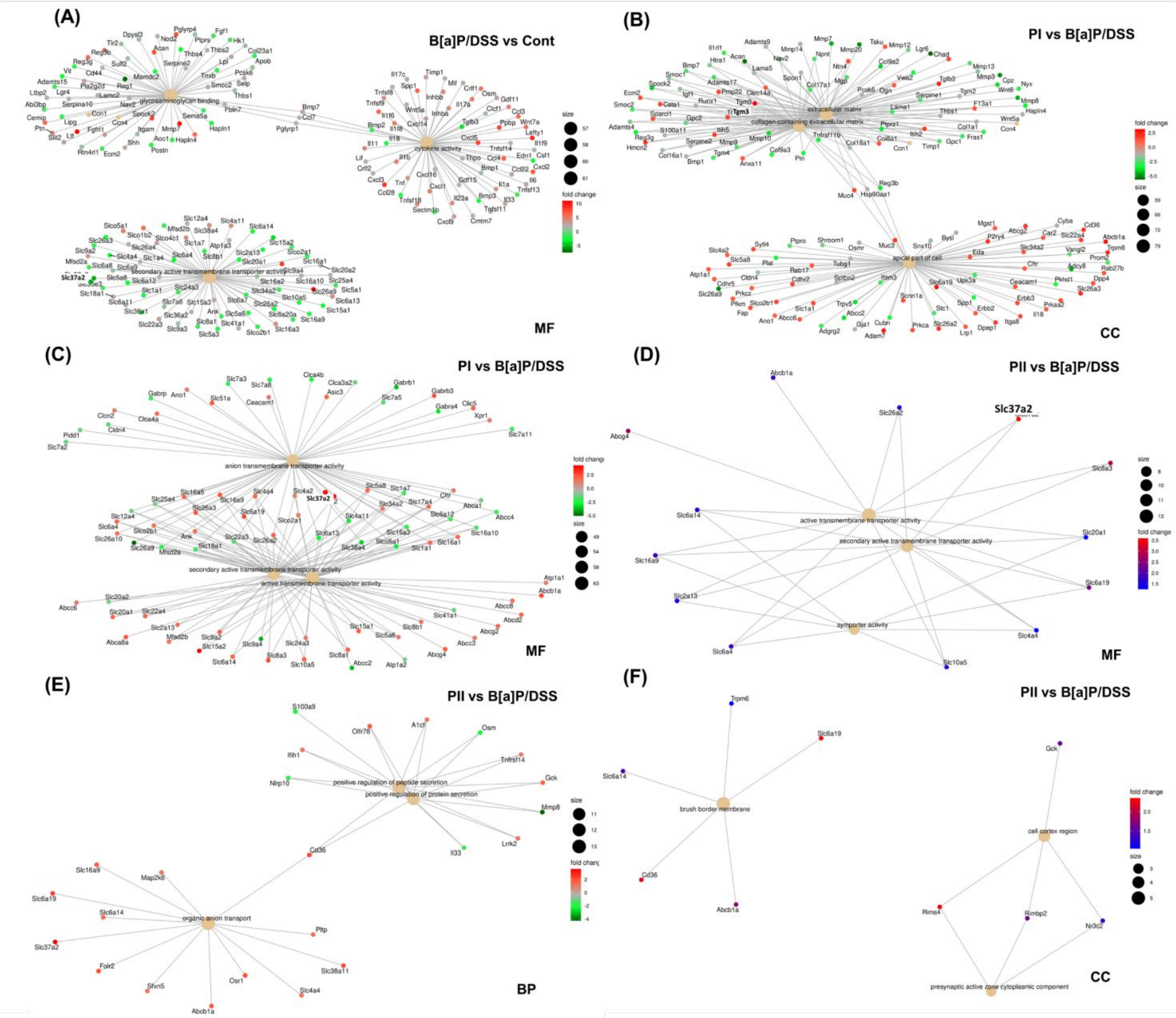

Supplementary Figure 5. Overview of GO modulated by B[a]P/DSS-induced colorectal cancer with different timing of PIC intervention.

(A) GO gene-concept network of core genes between B[a]P/DSS group vs Control group in Molecular Function (MF). (B) GO gene-concept network of core genes between PI group vs B[a]P/DSS group in CC. (C) GO gene-concept network of core genes between PI group vs B[a]P/DSS group in MF. (D) GO

gene-concept network of core genes between PII group vs B[a]P/DSS group in MF. (E) GO gene-concept network of core genes between PII group vs B[a]P/DSS group in BP. (F) GO gene-concept network of core genes between PII group vs B[a]P/DSS group in CC.

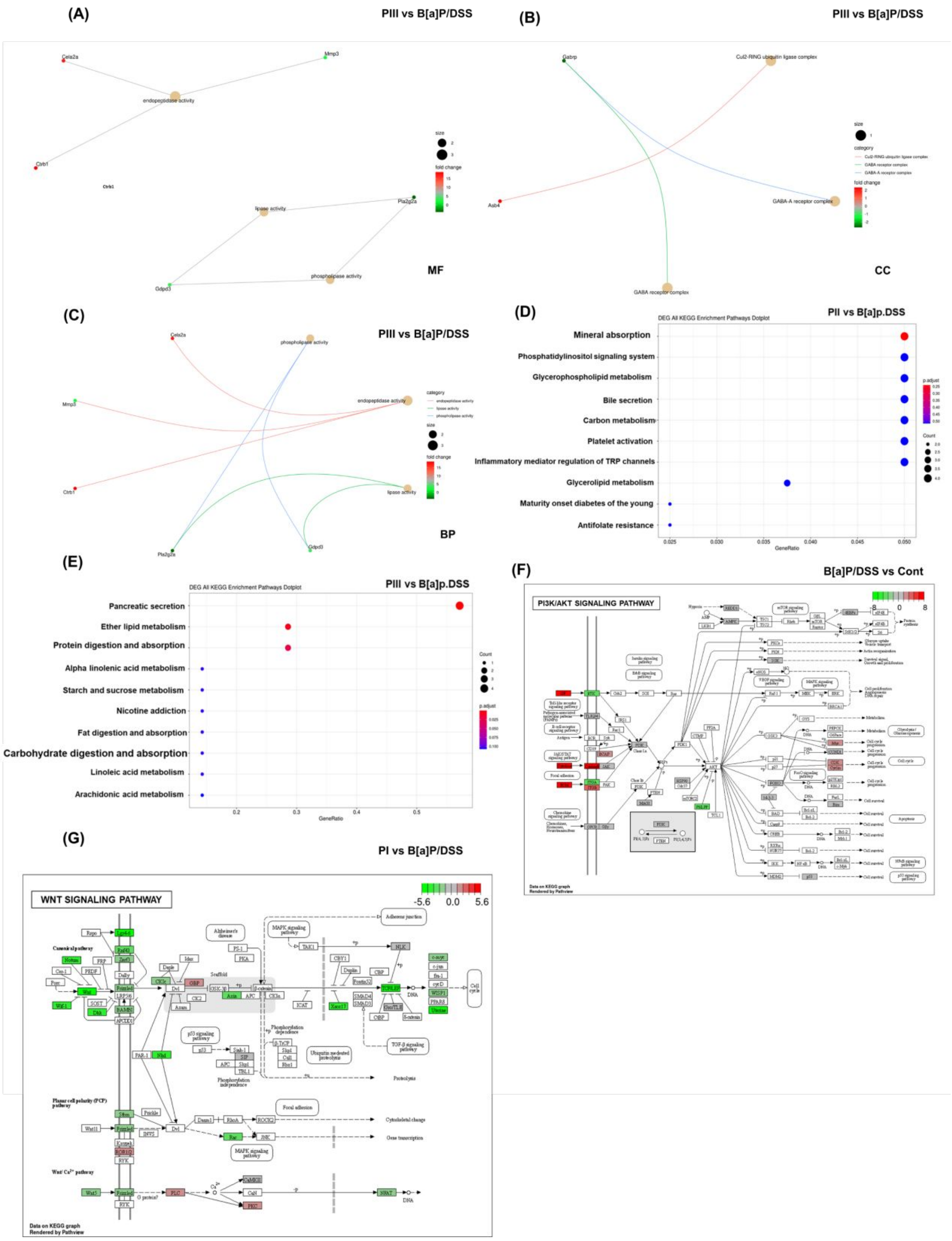

Supplementary Figure 6. Overview of GO and KEGG pathways modulated by B[a]P/DSS-induced colorectal cancer with different timing of PIC intervention. (A) GO gene-concept network of core genes between PIII group vs B[a]P/DSS group in MF. (B) GO gene-concept network of core genes between PIII group vs B[a]P/DSS group in CC. (C) GO gene-concept network of core genes

between PIII group vs B[a]P/DSS group in BP. (D) KEGG pathways dotplot between PII group and B[a]p/DSS group. (E) KEGG pathways dotplot between PIII group and B[a]p/DSS group. (F) PI3K/AKT signaling pathway in the KEGG pathway view of the gene map between the B[a]p/DSS group and control group. (G) WNT signaling pathway in the KEGG pathway view of the gene map between the PI group and B[a]p/DSS group. The enriched genes will be marked on the reaction pathway diagram, with red representing positive regulation and green representing negative regulation. n=3 per group.

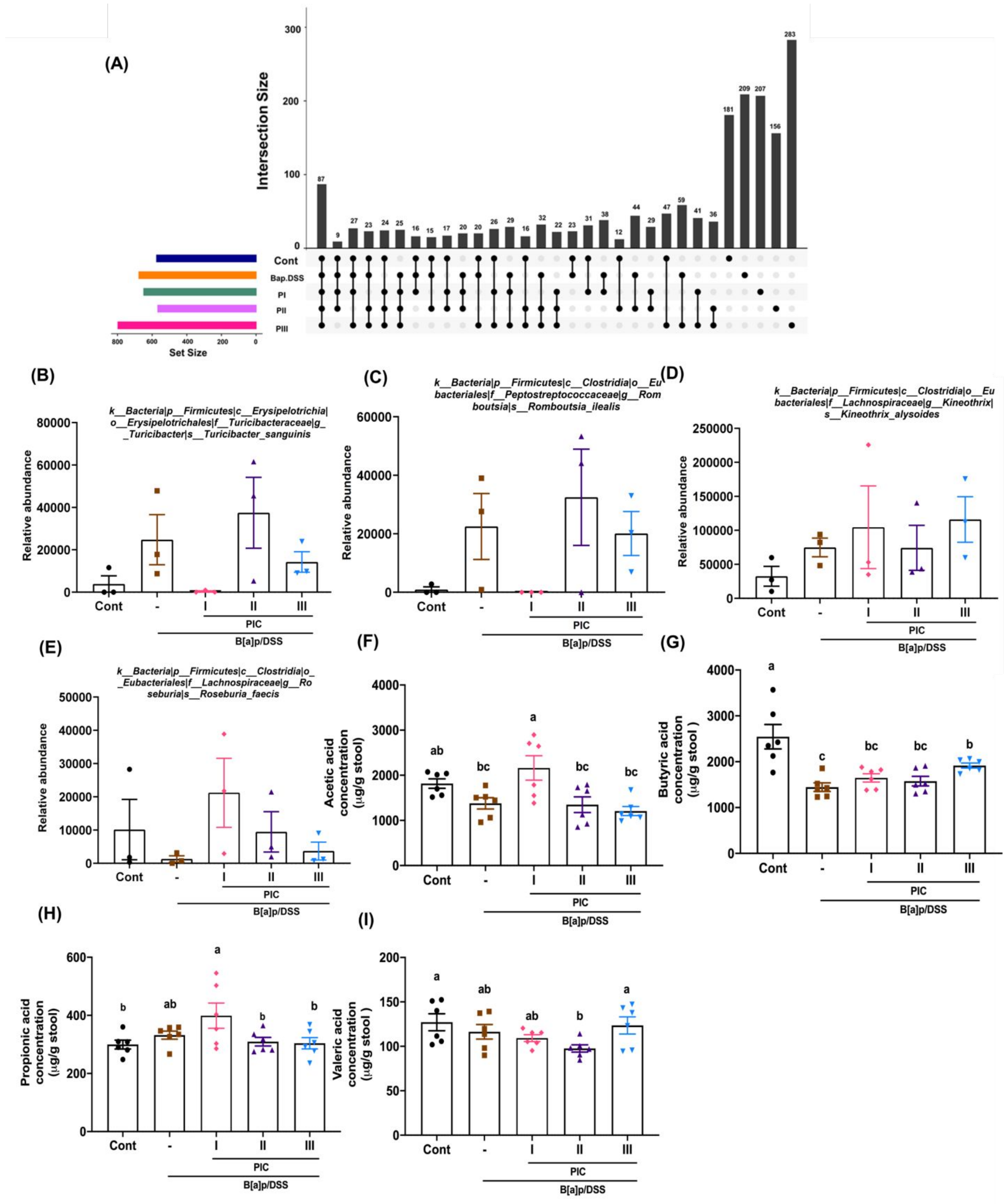

Supplementary Figure 7. Comparison of gut microbiota and fecal SCFAs levels among all experimental groups in B[a]P/DSS-induced colorectal cancer mice. (A) UpSet plot. Genus changes in gut microbiota: (B) *Bacteroides*. (C) *Candidatus_Stoquefichus*. (D) *Parabacteroides*. (E)

[Eubacterium]_coprostanoigenes. (F) *Alistipes*. (G) Acetic acid. (H) Propionic acid. (I) Butyric acid. (J) Isobutyric acid. (K) Isovaleric acid. (L) Valeric acid. Data are expressed as the means ± SEM. Values with different letters (a-c) differ significantly ($p < 0.05$) among the compared groups.
